# Combining evidence of selection with association analysis increases power to detect regions influencing complex traits in dairy cattle

**DOI:** 10.1186/1471-2164-13-48

**Published:** 2012-01-30

**Authors:** Hermann Schwarzenbacher, Marlies Dolezal, Krzysztof Flisikowski, Franz Seefried, Christine Wurmser, Christian Schlötterer, Ruedi Fries

**Affiliations:** 1 Lehrstuhl für Tierzucht, Technische Universität München, Hochfeldweg 1, 85376 Freising-Weihenstephan, Germany; 2 Institut für Populationsgenetik, Veterinärmedizinische Universität Wien, Veterinärplatz 1, 1210 Vienna, Austria; 3 ZuchtData EDV Dienstleistungen Ges.m.b.H. Dresdner Sraße 89/19 1200 Vienna, Austria

**Keywords:** selection signature, whole genome association, cattle, complex trait

## Abstract

**Background:**

Hitchhiking mapping and association studies are two popular approaches to map genotypes to phenotypes. In this study we combine both approaches to complement their specific strengths and weaknesses, resulting in a method with higher statistical power and fewer false positive signals. We applied our approach to dairy cattle as they underwent extremely successful selection for milk production traits and since an excellent phenotypic record is available. We performed whole genome association tests with a new mixed model approach to account for stratification, which we validated via Monte Carlo simulations. Selection signatures were inferred with the integrated haplotype score and a locus specific permutation based integrated haplotype score that works with a folded frequency spectrum and provides a formal test of signifance to identify selection signatures.

**Results:**

About 1,600 out of 34,851 SNPs showed signatures of selection and the locus specific permutation based integrated haplotype score showed overall good accordance with the whole genome association study. Each approach provides distinct information about the genomic regions that influence complex traits. Combining whole genome association with hitchhiking mapping yielded two significant loci for the trait protein yield. These regions agree well with previous results from other selection signature scans and whole genome association studies in cattle.

**Conclusion:**

We show that the combination of whole genome association and selection signature mapping based on the same SNPs increases the power to detect loci influencing complex traits. The locus specific permutation based integrated haplotype score provides a formal test of significance in selection signature mapping. Importantly it does not rely on knowledge of ancestral and derived allele states.

## Background

Linking genotype to phenotype is one of the central questions in biological sciences. Current approaches to map intraspecific variation to causative sequence variation use either a quantitative genetics framework (association mapping) or rely on population genetic theory (hitchhiking mapping).

Population genetic theory predicts that a favorably selected allele is either lost or increases in frequency until fixation [1]. With the spread of a beneficial allele, linked, non-selected sites also increase in frequency, a phenomenon that has been termed hitchhiking [1].

Based on this principle, genome scans were performed in a large number of species such as human, maize, Drosophila, *Arabidopsis thaliana *and *Plasmodium falciparum *[2-10]. Selection signatures in cattle based on SNP data on single chromosomes were reported on *Bos taurus *(BTA) chromosomes 6 [11], 19 [12] and 29 [13]. Barendse et al. [13], Gibbs et al. [14] and Hayes et al. [15] published genome wide maps of diversifying selection between *Bos taurus *dairy and beef cattle, Flori et al. [16] between three different French dairy cattle breeds, and Gautier et al. [17] among several West African cattle breeds. Qanbari et al. [18] employed an extended haplotype homozygosity test and published a genome wide map of recent selection within the German Holstein dairy cattle population. Gautier et al. [19] also used this signature of selection within a recently admixed Caribbean cattle breed. Furthermore these authors employed a modified version of Rsb scores proposed by [20] to detect local excess or deficiency from a given ancestry relative to the average genome admixture levels. Qanbari et al. [21] recently published a genome scan in several dairy and beef breeds including German Brown Swiss cattle based on integrated haplotype scores and when contrasting breeds employing F_ST _statistics. However, disentangling selection from nuisance signals caused by the demographic history of a breed or species based on genome wide polymorphism data remains challenging.

Stringent artificial selection resulted in an enormous improvement of production traits over the last couple of decades, especially for traits with moderate to high heritability. In combination with the availability of high density SNP arrays and high quality phenotypes, this intense selection renders the genome of dairy cattle an optimal model to look for signatures of recent positive selection.

While for genetic model organisms very powerful genomic tools are available, these species frequently lack phenotypic records to link signatures of selection in the genome to actual variation in phenotype unless a huge additional phenotyping effort is undertaken. This is the great advantage of using livestock species, as numerous production- and fitness traits are routinely recorded and used in breeding value estimation.

The estimated breeding value (EBV) expresses the genetic merit of a breeding animal estimated based on their own performance and performances of all available relatives. In the case of dairy bulls this typically includes hundreds to thousands of daughters. Furthermore EBVs are corrected for systematic environmental effects. Therefore the breeding value of an animal is the sum of its genes' additive effects based on Fisher's infinitesimal model [22], which assumes a very large (effectively infinite) number of loci each with very small effect. Although only approximatively correct, application of this model in selection paved the way for efficient livestock breeding.

Since Sax's experiments with beans in 1923 [23] we know however, that there are so called quantitative trait loci (QTL) that have a bigger than infinitesimal effect and that these loci can be mapped i.e. via linkage analyses. Such QTL mapping studies as a quantitative genetics approach have been very successful in cattle, see [24-26] for a summary.

Rapid improvements in high throughput SNP genotyping technologies and commercially available high density SNP arrays for livestock species allowed livestock geneticists to turn towards whole genome association (WGA) mapping approaches in the recent past e.g. [27-29] or see [30] for a review in livestock. The number of individuals that need to be genotyped to achieve reasonable power in a stand alone WGA is nevertheless still limiting [31].

Population genetics provides information that is independent of phenotypic information on putative loci under strong directional artificial or natural selection. We show in this paper that combining a population genetics signal with association tests based on quantitative genetics in a composite statistic, increases power and reduces the number of false positive signals for localizing the source of selection.

In a similar vein, [32] proposed a composite test statistic of several selection signature signals to increase power to detect selection. Barendse et al. [13] discussed the potential of combining genome wide scans for selection and whole genome association studies. However, as these authors were looking for signatures of diversifying selection based on F_ST _values the combination with association results is not straightforward. Akey at al. [33] followed up a region on dog (*Canis familiaris*) autosome 13 that showed evidence for selection in the Shar-Pei dog breed with association mapping and finally dissected the molecular basis of the typical skin wrinkling phenotype in this breed. Ayodo et al. [34] found that in a case-control candidate gene approach in humans the statistical power to detect disease variants can be increased by orders of magnitude by weighting candidates by their evidence of natural selection.

Our composite statistic combines a long-range haplotype statistic, based on genomic signatures of (new) positive mutations that are not yet fixed in a single population, and the regression coefficient based on allele-count indicator variables of a WGA - as the quantitative genetic approach. Both estimators rely on the underlying linkage disequilibrium (LD) between the causal variant and the genotyped SNP. We further propose a new mixed model approach to account for stratification in population based association studies, and we introduce a modified extended integrated haplotype score test statistic to detect selection. Using computer simulations and real data we show that the combination of both tests increases the power for localizing the target of selection relative to a single test and reduces the number of false positive signals.

## Methods

### Experimental Design

The highest selection pressure in the overall breeding goal in Brown Swiss cattle over the last decades was put on protein yield, the main trait of interest in this study to ensure high power for both mapping approaches.

The 140 highest and 148 lowest bulls with respect to protein yield EBV and a minimal EBV-accuracy (r^2^, degree of determination) of 0.9 were chosen out of 973 progeny tested Brown Swiss bulls for selective genotyping [35]. Up to two generations were present among the genotyped bulls. The bulls descend from 90 different sires and 121 maternal grandsires. Sire and maternal grandsire family size ranged from 1 to 20 and 1 to 34 members, respectively.

### Phenotypes

Sire EBVs were obtained from the genetic evaluation centre LfL Grub, Germany from the August 2008 genetic evaluation for PY. EBVs for protein yield are in kilogram units.

### Genotypes

Genomic DNA was prepared from semen straws following standard protocols using proteinase K digestion and phenol-chloroform. Across all samples the concentration was set to 50 ng/μl. Bulls were genotyped according to the manufacturer instructions with the Illumina BovineSNP 50K Bead chip^® ^comprising 54,001 SNPs at the Institute of Human Genetics of Helmholz Zentrum München, Germany. Genotypes of one individual were omitted due to a call rate of < 90%. The average call rate of the remaining 287 bulls was 98.6% corresponding to approximately 53,230 genotypes obtained per individual. The software PLINK, version 1.03 [36] was used to filter raw genotype data. SNPs with known genomic location on autosomes, with a minor allele frequencies of > 5%, that were missing in less than 10% of bulls were considered. We then filtered for all SNPs for which the ancestral state of the allele was reported by [37]. The final dataset contained 34,851 SNPs. Haplotypes were inferred with fastPHASE, version 1.2 [38]. Parameters in fastPHASE were set to 10 random starts for the EM algorithm and 10 clusters. Haplotypes were inferred for whole chromosomes ignoring pedigree information.

### Detection of Selection Signatures

We wrote R and C++ scripts to calculate extended haplotype homozygosity (EHH) test statistics from phased haplotype data as proposed by [6,7]. Briefly, the EHH of a core SNP is calculated as:

$$EHH_{i}=\frac{\overset{s}{\underset{j=1}{\sum }}\left(\begin{array}{c} e_{ij}\\ 2 \end{array}\right)}{\left(\begin{array}{c} c_{i}\\ 2 \end{array}\right)}$$

where c_i _is the number of samples of a particular core SNP allele i, e_ij _is the number of samples of a particular extended haplotype j, carrying the allele i at the core position, and s is the number of unique extended haplotypes [7]. EHH captures, as a function of distance, the decay of identity of haplotypes that carry a specific core allele. EHH starts at one and decays to zero, with increasing distance for both alleles at each core SNP. The area under the EHH curve that results from plotting EHH versus distance is expected to be greater for the selected allele than for the neutral allele. As proposed, we computed the integrated EHH (iHH) as the integral of the observed EHH in both directions from the core position until EHH reaches 0.05 [9]. The unstandardized integrated haplotype score (uiHS) is then calculated as

$$uiHS=\ln\left(\frac{iHH_{\text{ancestra}1}}{iHH_{\text{derived}}}\right),$$

Voight et al. [9] estimated the expectation and standard deviation (SD) of ln (iHH_ancestral_/iHH_derived_) in bins of derived allele frequencies from the empirical distribution at SNPs whose derived allele frequency *p *matches the frequency at the core SNP. The resulting standardized iHS (iHS^Voight^) follows approximately a standard normal distribution.

$$iHS^{Voight}=\frac{uiHS-E_{p}\left(uiHS\right)}{SD_{p}\left(uiHS\right)}$$

Since standardisation is based on the frequency of the derived allele this sets an upper limit to the age of the mutation. This test statistic answers the question of how unusual the length of a haplotype is, assuming the same age of allele across all observed selection coefficients acting on any core SNP with a similar derived allele frequency in the genome. It therefore does not provide a formal test of significance. Furthermore if different outgroups are used to define ancestral and derived states this sets different age boundaries to the mutations resulting in less precise standardisation.

### A locus specific permutation-based iHS

When the rate of EHH decay is similar for the ancestral and derived allele, as expected for a neutral locus, uiHS is ~ 0 [9].

Voight et al. [9] showed via simulation that extremely positive and negative iHS scores are both potentially interesting selection signals and polarisation with the ancestral allele results in a change of sign, but does not change the magnitude of the uiHS test statistic.

In the following we introduce a locus specific permutation based approach that relies on minor and major allele frequencies rather than ancestral and derived states, respectively. Most importantly this test statistic provides significance of deviations of uiHS from its neutral expectation.

The core site in iHS test statistics is used to define two groups of haplotypes for comparison with regard to their block structure. We shuffled core SNP alleles 1,000 times at the core position while retaining the neighbouring haplotype configuration and calculated uiHS for each permuted sample (iHS_P_) within each core SNP. Random shuffling SNP alleles at core sites randomizes allocation of haplotypes to the two groups for comparison while maintaining the LD structure in the surrounding genomic region. This simulates the null hypothesis of neutrality: the site was not subject to selection. We hereby obtain an empirical distribution of iHS under the H0 for each SNP, from which we obtain the probability that we see such an extreme iHS just by chance. The locus specific standard deviation of the 1,000 iHS_P_ test statistics is then used to scale the observed deviation of uiHS from its expectation zero. Scaled, permutation based iHS (siHS_P_) is therefore calculated as

$$siHS_{P}=\frac{uiHS-0}{SD\left(iHS_{P}\right)}$$

Since the empirical mean of permuted iHS statistics is approximately 0 (see Additional file 1, Figure S1) our test is a formal test of significance, given the allele frequency of the core site and the LD structure in the surrounding region. This is a property of crucial importance of a test statistic, especially since we want to combine our results with an association test from a WGA study.

Generally SNP sites with low minor allele frequencies show larger SD of iHS_P_. However, to avoid any additional bias due to possibly remaining dependence of siHS_P _on allele frequency, siHS_P _was fit in a linear model by regressing the SNP minor allele frequency (MAF_i_) at the core site on siHS_P_. For each site, the random residual ε_ij _was obtained and subsequently standardized using the standard deviations SD(ε_ij_) of residuals across all SNPs. In contrast to [9] (here termered as iHS^Voight^), our frequency correction is not done based on the expectations of SNPs within allele frequency bins but carried out on a continuous scale.

$$siHS_{P\;ij}=MAF_{i}+\varepsilon_{ij}$$

The resulting frequency corrected, and scaled test statistic is termed iHS.

$$iHS=\frac{\varepsilon_{ij}}{SD\left(\varepsilon_{ij}\right)}$$

This final test statistic is approximately standard normally distributed.

Since no high resolution genetic map was available for the SNPs in this study, physical distances between SNPs were used for calculating all integrated haplotype scores.

### Whole Genome Association Study

Standard statistical tests, e.g. regressing phenotype on allele count in a linear model, are inappropriate for population based WGA in structured populations because they either result in an inflated proportion of spurious marker - phenotype associations or mask true associations (e.g.[39,40]), even with modest levels of population stratification and/or admixture. In the case of cattle populations, artificial insemination schemes allow the use of a few superior bulls as sires of the next elite sire generation. Thus, genotyped bulls are frequently paternal half-sibs or share the same maternal grandsire. The subsequent family structure can cause substantial stratification. Quantile - quantile plots and inflation factors (*λ*) were used to characterize the extent to which the observed distribution of *P*-values follows the expected null distribution. Inflation factors were calculated as

$$\lambda =Median\left(T_{1}^{2},T_{2}^{2},\ldots T_{N}^{2}\right)/0.456$$

with $T_{i}^{2}=\beta_{i}^{2}/Var\left(\beta_{i}\right)$, where *β_i _*is the effect of the i-th SNP (i from 1 to N),Var(*β_i_*) the variance of the estimate and 0.456 the median of the $\chi_{1df}^{2}$ distribution [41].

Recently, linear mixed models were proposed to effectively account for different levels of relatedness by incorporating pairwise genetic relatedness into the model [31]. This approach relies on the fact that the phenotypes of two genetically related animals are more similar than those of genetically distant individuals. Estimation of covariance between individuals is assisted by the availability of a marker based kinship matrix, which can be estimated more accurately using genotype data from the WGA experiment than from pedigree information.

We therefore employed the following single locus mixed model which we term "MIX" that explicitly models the polygenic relationships among inviduals, as

(1) y=Xb+Za+e,

where y is a vector of sire EBVs for protein yield, X is the design matrix in which SNP genotypes were coded 0, 1 and 2, counting the number of minor alleles and b the vector of regression coefficients on recoded SNP genotypes. Z denotes the design matrix for random effects with a ~ N (0, **G**σ_a_^2^) being the vector of polygenic effects, σ_a_^2 ^the additive genetic variance and **G **the genetic covariance matrix and e ~ N (0, **I**σ_e_^2^), a vector of residual effects. **G **was obtained from pairwise identical by descent (IBD) estimates using genome wide SNP data as implemented in PLINK [36], in which the IBD state is estimated by a hidden Markov model, given the observed identity by state (IBS) sharing and genome wide levels of relatedness between the pairs. Diagonal elements of **G **were calculated as 1+F, with *F *being the inbreeding coefficient estimated from SNP data using PLINK [36].

Mixed models were solved in R (http://www.cran.r-project.org) via direct matrix inversion. Empirical *P-*values were calculated by an adaptive permutation procedure, shuffling the vector of genotype codes among phenotypes. This does not destroy the relationship between IBD status and phenotypes, but breaks up any association between SNP genotypes and phenotypes. This leaves LD patterns unperturbed and hence does not control for stratification. The number of permutations was sequentially increased up to 1 × 10^6 ^permutations if the SNP indicated association. The empirical *P-*value was calculated as the number of test statistics obtained on permuted sets being greater than or equal to the observed test statistic.

All 34,851 SNPs were tested one after the other for association with the protein yield (PY) phenotype.

As model MIX did not overcome the stratification present in our highly structured sample we applied a two stage approach. Besides accounting for the relationship via a mixed model, stratification was accounted for by pre-correcting SNP genotype codes for sire and maternal grandsire (MGS) differences using the following regression model

(2) $$gt_{ij}=sire_{j}+MGS_{j}+\varepsilon_{ij},$$

where gt is the recoded genotype code (0, 1 and 2 for 1-1, 1-2 and 2-2 allele combinations, respectively with 1 representing the minor allele), sire is the fixed effect of sire *i *and maternal grandsire the fixed effect of maternal grandsire *j *and ε_ij_~ N (0, **I**σ_e_^2^), the vector of random residual effects. Sire- and maternal grandsire families smaller than five were merged into one group.

Residuals ε*_ij _*were used instead of raw recorded genotypes (0, 1 and 2) in the design matrix X of equation (1), henceforth termed method "MIXStrat".

### Evaluation of WGA via Monte Carlo Simulation

The proposed method to account for stratification is specific to situations typically observed in intensively selected livestock species and populations. We evaluated the effectiveness of MIXStrat by Monte Carlo simulations. Phenotypes, sire- and maternal grandsire family structure were taken from the population under consideration. Genotypes for 287 bulls and 10,000 diallelic sites were sampled based on the following procedure:

First, the allele frequency *p *of the first allele at a SNP was drawn from a uniform distribution, the allele frequency for the second allele *q *at this SNP is then given by *q = 1-p*. Two alleles each were sampled for all sires and maternal grandsire according to these frequencies. Bulls inherited sire and maternal grandsire alleles following Mendelian rules. Alleles inherited via the dam were sampled corresponding to the population allele frequencies. This simulates the null model (e.g. no effect of the locus on the phenotype) taking into consideration the observed population structure. Association was tested for, using the models MIX and MIXStrat.

### Rate of False Positives

The average rate of false positive detections across m = 100 random repetitions was calculated as

$$\alpha_{P}=\overset{m}{\underset{i=1}{\sum }}\left(\frac{P\in \left\{P<0.05\right\}}{10,000}\right)/m$$

### Power Analysis

For true associations the mean genotype values within bulls of sire and maternal grandsire are correlated with phenotypic family means. This information is not utilized when genotypes are recoded and will thus reduce power. We evaluated the power of MIXStrat relative to the power of MIX under the alternative model. This was achieved by simulating an additive QTL effect which explained 1, 5 and 10% of the EBV variance:

$$\alpha_{QTL}=\sqrt{\frac{\sigma_{EBV}^{2}QTL_{SIZE}}{2p\left(1-p\right)}}$$

where α_QTL _is the allele substitution effect [42], σ^2^_EBV _the variance of EBV, QTL_SIZE _is the size of the effect as proportion of σ^2^_EBV _and *p *the allele frequency of the simulated diallelic locus. Power was calculated as

$$Power=\overset{m}{\underset{i=1}{\sum }}\left(\frac{P\in \left\{P<\alpha_{Bonf}\right\}}{10,000}\right)/m$$

with α_Bonf _being the 5% Bonferroni- corrected type I error threshold of 2.5 × 10^-5 ^and m being the number of random Monte Carlo repetitions.

### Composite Test Combined Significance Test and False Discovery Rate

We used Stouffer's method [43] to combine *P- *values from the association study with those from the selection signature analysis (*P_COMB_*).

The test statistic was calculated as

$$Z=\overset{k}{\underset{i=1}{\sum }}z\left(P_{i}\right)/\sqrt{k}$$

where Z is the standard normal variable under H_0_, z(P_i_) is the *P *- value from test *i *transformed to Z and k is the number of tests that are combined in the test statistic. *P *- Values P_COMB _were obtained using the quantile function of the standard normal distribution. The tail area based false discovery rate (FDR) was calculated from *P*_COMB _values using the R package fdrtool, v1.2.5 [44]. Significance was declared if the *q *value (FDR corrected *P - *Value) was < 0.10.

## Results

### Evaluating the locus specific permutation of the iHS test statistic to detect signatures of selection and comparison to iHS^Voight^

We mapped selection signatures with iHS^Voight ^and our newly proposed iHS to detect sites under selection.

Table 1 shows the expectations and standard deviations for each of the derived allele frequency bins used for the frequency correction to calculate the approximately standard normally distributed iHS^Voight ^test statistic. Differences in expectations among derived allele frequency bins (Table 1) necessitate working with an unfolded frequency spectrum for iHS^Voight^.

**Table 1:** Means and standard deviations (SD) in defined frequency bins for uncorrected integrated haplotype score (uiHS) test statistics to calculate iHS^Voight^.

Frequency of derived allele	Mean	SD
<= 0.1	-1.04	1.04
0.1 - 0.2	-0.93	0.94
0.2 - 0.3	-0.73	0.92
0.3 - 0.4	-0.48	0.92
0.4 - 0.5	-0.26	0.93
0.5 - 0.6	-0.06	0.92
0.6 - 0.7	0.16	0.94
0.7 - 0.8	0.39	0.95
0.8 - 0.9	0.65	0.99
> 0.9	0.75	1.06

**Figure 1:**
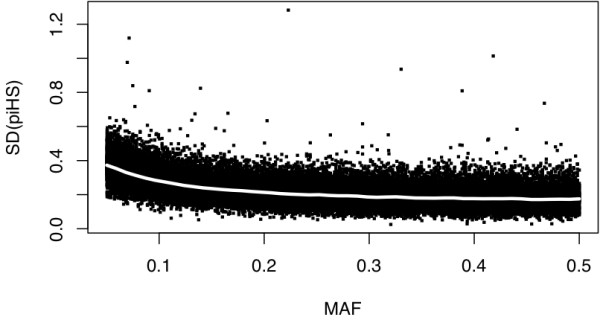
**Plot of standard deviations of permuted integrated haplotype scores (iHS) (1,000 permutations) versus minor allele frequency (MAF)**.

Figure 1 shows that the standard deviation of 1,000 randomly permuted iHS statistics (iHS_P_) is nearly constant at ~0.18 for SNPs with a MAF > 15% but increases more than two fold for SNPs with lower MAFs. A similar trend can be seen for iHS^Voight ^where SD in the <= 0.1 and > 0.9 is higher compared to the rest of the derived allele frequency bins.

For SNPs with low minor allele frequencies we found a relatively higher proportion of extreme unscaled iHS statistics. We postulate that this is due to increased rates of false positives, since power simulations by [9] and [45] show that iHS^Voight ^is powerful for loci with intermediate allele frequencies and that the power of the test drops substantially when the selective sweep is close to fixation, in other words for SNPs with low MAF.

**Figure 2:**
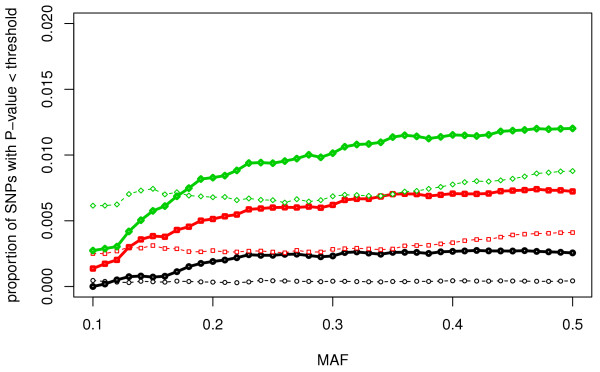
**Proportion of SNPs with significant selection signals, relative to all SNPs with a minor allele frequency (MAF) below the value given along the x-axis**. solid line: permutation based iHS, dashed line: iHS^Voight^; symbols: circles, squares and rhombs symbolize SNPs with P- values for the corresponding test statistic below 0.001, 0.005 and 0.01, respectively.

Figure 2 shows that the proportions of permutation - based iHS signals with a *P *- Value < 0.001, < 0.005 and < 0.01 are relatively smaller for SNPs with low minor allele frequency. This tendency cannot be seen as clearly for the iHS^Voight ^test statistic. For SNPs with MAF ≥ 0.20 we see that our iHS test yields a higher proportion of significant loci when compared to traditional iHS^Voight^.

**Figure 3:**
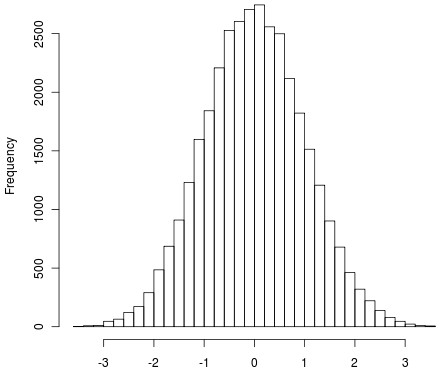
**Histogram of iHS^Voight ^test statistics from selection signature analysis for 34,851 SNPs**.

**Figure 4:**
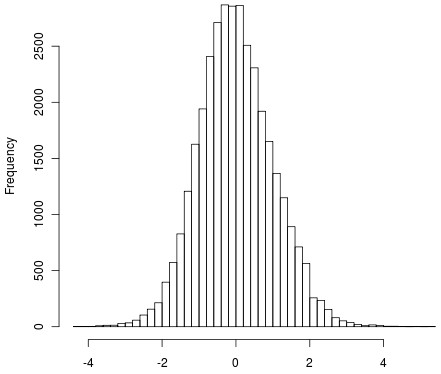
**Histogram of iHS test statistics from selection signature analysis for 34,851 SNPs**.

**Figure 5:**
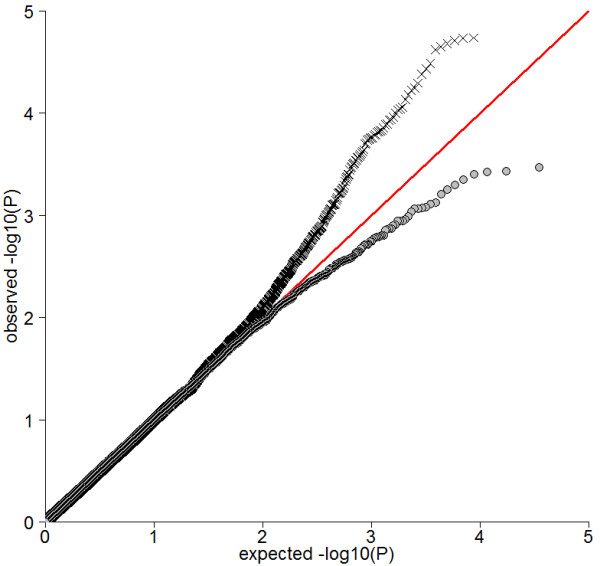
**Quantile - quantile plot of *P *- Values from selection signature analysis for 34,851 SNPs using our modified iHS (X) and the iHS^Voigth ^(●) test statistics, respectively**.

Figures 3 and 4 show the histograms of iHS^Voight ^and iHS, respectively. Figure 5 shows a QQ-plot of iHS and iHS^Voight^.

Figures 3, 4 and 5 all suggest that iHS has increased power compared to iHS^Voight^.

Our permutation based standardization allows a formal test against the null hypothesis of neutrality at a core SNP (expectation zero). Our standardization is against 1000 permuted test statistics at the same locus in the same LD background. We therefore do not need to define the state of ancestral and derived allele.

Additional file 1, Figure S2 shows a histogram of derived allele frequencies and Additional file 1, Figure S3 a histogram of minor allele frequencies of the 34,851 SNPs used in this study. Additional file 1, Figures S4 and S5 show histograms of *P *- Values for iHS^Voight ^and iHS, respectively.

### Detection of Selection Signatures in the Brown Swiss dairy cattle population

Manhattan plots for iHS^Voight ^and iHS for each autosome except BTA 6 are shown in Additional file 2, Figure S6 - S33 plots A and B.

Among the 34,851 SNPs tested genome wide 1,710 and 1,621 SNPs had a test statistics > |1.96| with method iHS^Voight ^and iHS, respectively.

Distribution among chromosomes is remarkably uneven: BTA 5, 6, 12, 19 harbor 148, 124, 98, 89 sites, respectively which corresponds to 8 - 11% of all investigated SNP on the corresponding chromosomes that show significance applying iHS. On other chromosomes, namely BTA 28 and 17 ~ 1% of investigated SNPs exhibit significant selection signatures.

The same is true for iHS^Voight ^BTA 5, 6, 12, 16 and 19 have 171, 131, 148, 136 and 112 SNPs that show an iHS^Voight ^test statistic > |1.96| which corresponds to 8 - 14% of all SNPs on these chromosomes. BTA 7, 25 and 27 have only around ~ 1% sites with extreme iHS^Voight ^test statistics.

**Figure 6:**
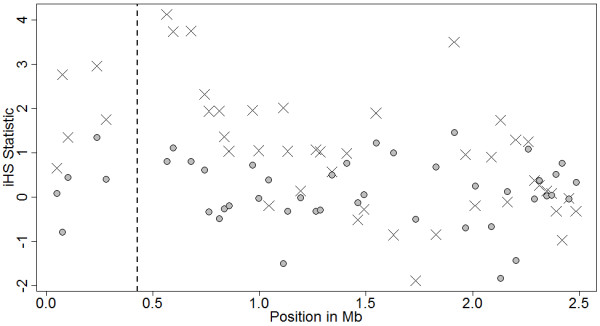
**iHS (X) and iHS^Voight ^(●) on proximal end (0 to 2.5 Mb) of BTA 14**. The vertical line marks the position of DGAT1 K232A locus. The x-axis displays the physical position in megabases.

**Figure 7:**
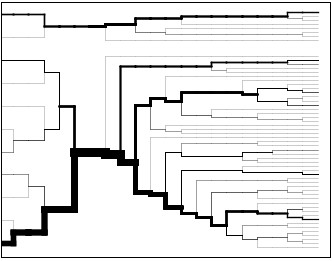
**Haplotype bifurcation plot of Hapmap52798-ss46526455**. The top figure shows the sweeping allele "G" while the bottom figure shows allele "A". This figure shows the breakdown of LD from the core SNP with increasing distance in both directions. The core SNP represents the root of the diagram. Each SNP represents a node and is an opportunity for further branching. If both alleles of a SNP are present on a haplotype the line branches. The thickness of the lines corresponds to the number of samples carrying the haplotype. The length of a branch corresponds to the distance between SNPs.

**Figure 8:**
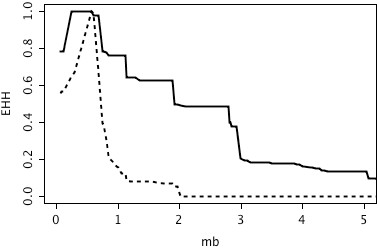
**Plot of EHH statistics of minor allele "G" (solid line) and major allele "A" (dotted line) of Hapmap52798-ss46526455 on proximal end of BTA 14**. The x-axis displays the physical position in megabases.

One particularly illustrative example is given by SNP Hapmap52798-ss46526455 located in the proximal region of BTA 14 at 0.565311 Mb (see Figures 6, 7 and 8 and Additional file 2, Figure S18). An iHS of 4.13 for this SNP with a frequency of 0.2 for allele G exhibits a larger area under the EHH curve as compared to allele A and was possibly under selection. Interestingly, this SNP is in close neighborhood to the well known *DGAT1 K232A *polymorphism, located at 0.444-0.447 Mb, with strong effects on milk production traits (e.g. [46-50]). In the Brown Swiss (BS) breed the frequency of the K allele is very rare with about 2% in the German BS population [48] and fixed for the A allele in the Italian BS population [51]. Most likely allele A was selected for because of its milk yield increasing effect while it reduces fat content. Note that the *DGAT1 *K to A mutation itself is not part of the Illumina BovineSNP 50K Bead chip^®^. Interestingly, our iHS provided a strong and convincing signal of selection, while the iHS^Voight ^(0.81) provides considerably weaker support. Hence, this might illustrate the increased power of our modified iHS as compared to the iHS^Voight^.

However, there is growing evidence for additional polymorphisms in the *DGAT1 *gene and its neighborhood that cause phenotypic variation for milk production traits eg [52,53]. Of particular interest is a QTL mapping study in the German-Austrian-Italian BS population [54], that reported significant QTL for milk yield and protein percent in the *DGAT1 *region although all bulls in this study were shown to be homozyogous for the p.K232A polymorphism [55]. This finding is supported by the large SNP effects estimated for fat and protein percent in the US - BS population (http://aipl.arsusda.gov/Report_Data/Marker_Effects/marker_effects.cfm?Breed=BS) albeit the near fixation of allele A in this breed. So it is likely that the selection signal that is picked up by iHS is not purely for the *DGAT1 *p.K232A polymorphism but for the proximal region of BTA14 as a whole including the VNTR polymorphism in the promoter region of the *DGAT1 *reported by [53].

### Association Study on PY

**Figure 9:**
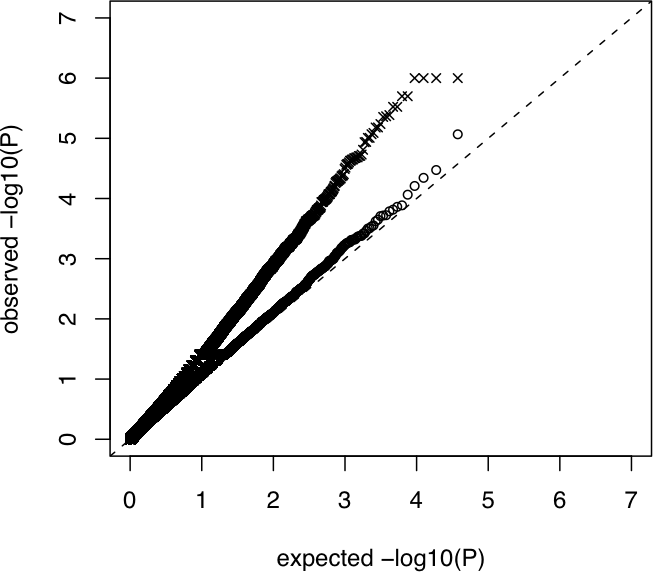
**Quantile - quantile plot from association study on protein yield using model MIX (✘) and MIXStrat (Ο), respectively**.

We used 34,851 SNPs that met our stringent quality criteria and also had the ancestral allele reported in literature for association testing. Population stratification was accounted for by including IBD estimates from the genotype data (method MIX). A quantile - quantile plot analysis indicated, that this procedure did not sufficiently account for population stratification in our dataset (inflation factor λ = 1.34) (Figure 9).

We therefore developed a new strategy to reduce the number of erroneous association signals in our data (method MIXStrat). Both the quantile - quantile plot (Figure 9) as well as an inflation factor *λ *of 1.02 confirmed that the MIXStrat model successfully controlled for spurious results caused by stratification of our sample. Nevertheless, we also experienced a drop in power, as expected. The SNP with the smallest *q *value in method MIXStrat was 0.5639561 (tail-area based false discovery rate (FDR)) calculated with R package fdrtool, v1.2.5 [44], corresponding to a nominal P-value of 4.778973e-04. Note that the flattening out of the *P *-Value curve for method MIX is a consequence of the adaptive permutation procedure.

### Evaluation of WGA via Monte Carlo Simulation

Computer simulations showed that using MIXStrat the sample size in this study is sufficient to only detect strong effects explaining at least 10% of the phenotypic variation. The Monte Carlo simulation did not account for LD because conservative significance thresholds using Bonferroni correction were used. Nevertheless, it assesses the influence of population substructuring in single SNP regression whole genome association studies. Our simulations show clearly that the sire-, paternal grandsire- and maternal grandsire structure in dairy cattle populations alone can create significant results without any association between genotype and phenotype.

Additional file 3, Figure S34 shows a histogram of allele substitution effects across all 34,851 SNPs tested.

### Rate of False Positives

**Figure 10:**
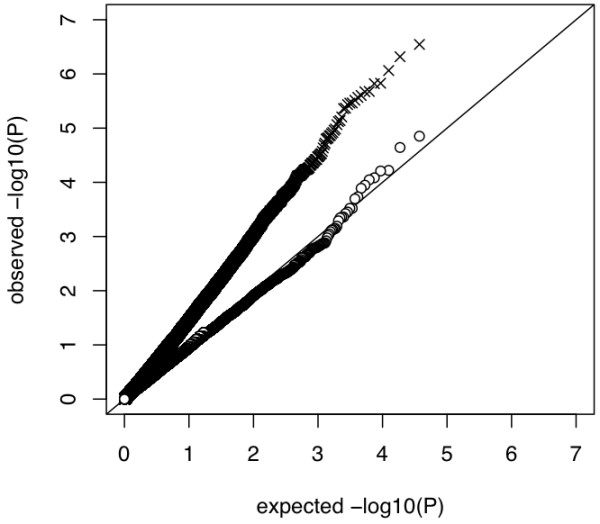
**Quantile - quantile plot for protein yield under the null distribution from method MIX (✘) and MIXStrat (Ο)**.

Empirical type I error rate *α *and the inflation factor *λ *using MIXStrat were 0.05 and 0.99, respectively, while an *α *of 0.13 and *λ *1.35 was observed when method MIX was applied. Furthermore the quantile - quantile plot in Figure 10 shows clearly that a standard mixed model cannot fully account for the stratification present in the data, whereas our MIXStrat approach succeeds in controlling the type I error rate under the simulated null distribution.

### Power Analysis

As expected, MIXStrat reduced power under the model of an existing QTL (Table 2). Power reduction was 0.069 and 0.10 for QTL that explain 10 and 5% of the trait variance, respectively. The relative power loss compared to MIX analysis of 8.6 (10% QTL size) and 30.6% (5% QTL size) indicates, that for large and particularly moderate QTL sizes our method leads to substantial power reduction.

**Table 2:** Results from power calculations of the Monte Carlo simulation; the underlying models of MIX and MIXStrat are described in the "algorithm section" of the paper.

QTL size in EBV variance	MIX	power of MIXStrat
1%	0.026	0.005

5%	0.347	0.234

10%	0.772	0.727

As further shown in Table 2 our dataset has sufficient statistical power to detect QTL explaining > 10% of the variance in EBV. Effect sizes of that magnitude are expected to be rare in livestock species [56]. MIXStrat without integration of information on selection signatures has insufficient power to detect loci explaining only 1% of the variance.

### Consensus of Selection Signature Signals and Association Signals

A positive iHS value indicates that the minor SNP allele, relative to the major allele, is associated with the larger integrated EHH statistic and was possibly selected for. Likewise the estimated regression coefficient in the association analysis (β_MIXStrat_) represents the estimated increase in trait value per additional copy of the minor allele. Thus alike signs of iHS test statistics and β_MIXStrat _indicate that the SNP is causative by itself or is in LD with a causative site that is under positive selection. Opposite signs of iHS and β_MIXStrat _may be observed when sites have pleiotropic effects and were selected on a different, possibly unobserved, trait. Generally one would expect to see a higher proportion of like signs as compared to opposite signs and a positive correlation coefficient for traits of major economic importance in the selection history of a breed.

Table 3 shows the correlations between iHS test statistics and allele substitution effects given by b_MIXStrat _for PY based on all 34,851 sites, as a quantitative evaluation of accordance. As expected the overall correlations among all sites was low. The correlations between allele substitution effects and iHS among sites identified to be under selection however was substantial with 0.466 among the top 1% of sites and even higher among the top 0.1% of sites. IHS^Voight ^however was uncorrelated with top 1% sites and showed a lower correlation of 0.228 among the top 0.1% sites as compared to iHS. This further supports our notion that iHS is an improved haplotype based test statistic for identifying important loci.

**Table 3:** Pearson correlation coefficients (95% confidence intervals) of different iHS statistics with regression coefficients from association study for protein yield.

Method	SNPs with MAF < 10%			all SNPs	
	all (N= 4,387)	top 1% |iHS| (N = 42)	all (N = 34,851)	top 1% |iHS| (N = 349)	top 0.1% |iHS| (N = 35)
iHS	0.045	0.197	0.091	0.466	0.559
	(0.016-0.074)	(-0.105-0.467)	(0.080-0.101)	(0.380-0.544)	(0.277-0.751)
iHS^Voight ^[9]	0.005	0.21	-0.005	0.002	0.228
	(-0.025-0.034)	(-0.092-0.31)	(-0.0158-0005)	(-0.107-0.102)	(-0.114-0.521)

### Combining Signatures of Selection with Association Tests

**Figure 11:**
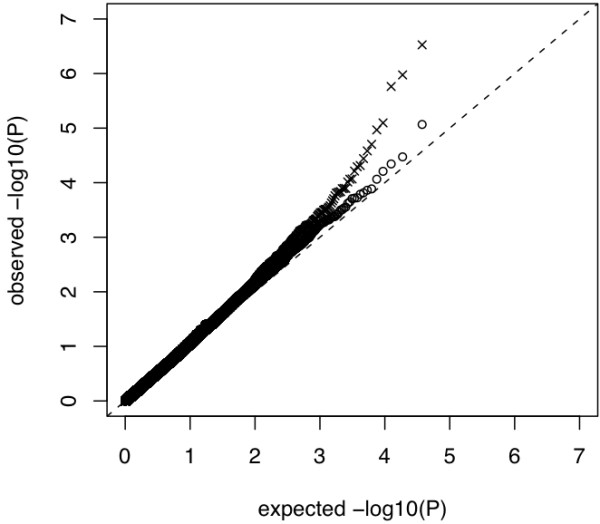
**Quantile - quantile plot from association study on protein yield using model MIXstrat (**Ο**) and combined test of selection signature iHS test statistics and whole genome associations with model MIXstrat (✘)**.

Selection signature - and association test statistics were moderately correlated (Pearson correlation coefficient was 0.091 for iHS and -0.005 for iHS^Voight^) across all 34,851 SNPs as the majority of SNPs are not in LD with a causative locus and therefore not under selection. This justifies treating the two sets of results as independent and using Stouffer's method to obtain *P- *Values (*P_COMB_*) from a combined significance test. Figure 11 indicates that combination of tests increases power of detection substantially. We applied a FDR threshold of 0.10, which corresponds to a nominal *P*-value cut-off of 2.149935e-06.

Additional file 2, Figures S6 - S33 show Manhattan plots for each of the bovine autosomes, combining model MIXstrat with iHS^Voight ^(plot C) and MIXstrat with iHS (plot D). All Manhattan plots are annotated with selection signature signals among the top 5% found by [21] applying iHS^Voight ^in windows of 500 kB in BS cattle (symbol o) and in any of the other breeds investigated, symbol (x). All plots are further annotated with QTL results reported from whole genome association studies in the cattle QTL database "Cattle QTLdb" [57]. We downloaded the gff3 file for btau4 at [57,58]http://www.animalgenome.org/cgi-bin/QTLdb/BT/download?file=gbpBTAU. QTL positions are annotated at the midpoints between start and end position of the reported QTL. QTL annotated outside the assembled bovine autosomes and in reverse direction (end position further distal than start of QTL) were filtered. Capital letters summarize QTL trait ontology classes: B for meat (beef) traits, E for exterior traits, H for health traits, M for milk traits, P for production traits, R for reproduction traits as classified at animalgenome.org

Only QTL annotated from WGA studies were considered, because of the large confidence intervals of QTL positions from linkage studies.

Additional file 3, Figure S35 shows a histogram of Stouffer's *P *- Values combining whole genome association results with model MIXstrat and iHS^Voight ^while Additional file 3, Figure S36 a histogram of Stouffer's *P *- Values combining whole genome association results with model MIXstrat and iHS.

**Figure 12:**
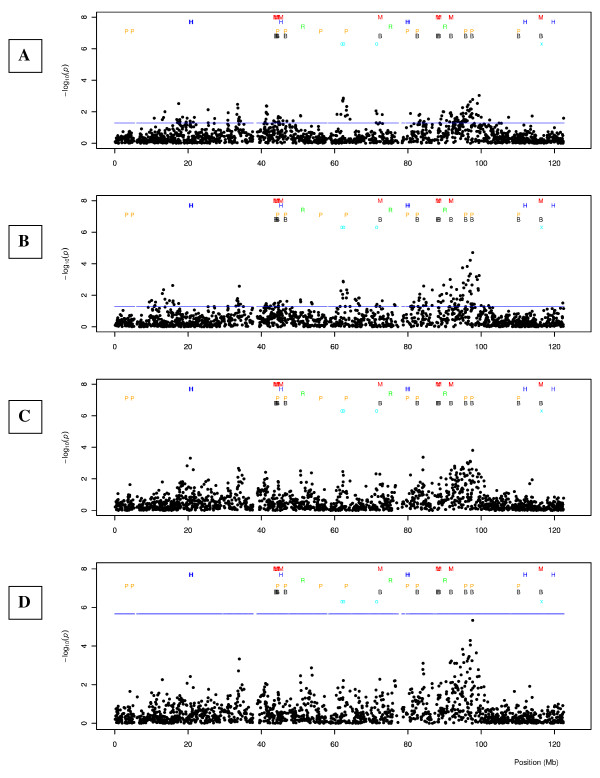
**Manhattan plots of chromosome 6**. Legend Figure 12: Capital letters denote QTLs reported from whole genome association studies (WGA) at [58], summarized as QTL trait ontology classes: B.. meat traits, E... exterior traits, H.. health traits, M.. milk traits, P.. production traits, R.. reproduction traits; o annotates a top 5% iHS^Voight ^test statistic as reported in by [21] in windows of 500 kb in Brown Swiss, × in any of the other breeds investigated; Plot A: iHS^Voight ^test statistics, blue line: threshold identifying the top 5%; B: iHS test statistics, blue line: threshold identifying the top 5%; C: combined iHS^Voight ^and WGA results with model MIXstrat, D: combined iHS and WGA result with model MIXstrat; blue line is a at 10% false discovery rate threshold.

Plots A and B in Figure 12 report selection signature mapping results applying method iHS^Voight ^and iHS, respectively. We see a nice agreement for both test statistics with the selection signatures reported by [21], in the same breed around 60 and 70 Mb. Both plots show an additional strong signal for selection in the region between 80 - 100 Mb which harbours the well studied casein gene family. This becomes evident by the large number of annotated WGA results in this region. Recently [59] reported a long range haplotype affecting protein yield and mastitis susceptibility in Norwegian Red cattle that was introgressed from a Swedish Holstein bull into Norwegian Red. SNPs in this region almost reach significance in the combined approach (plot D), which clearly demonstrates the increased power of the combined approach, as results from stand alone WGA applying model MIXstrat was far from signifance for any of the tested SNPs.

Hayes et al. [11] do not provide a supplemental table of iHS test statistics, we could therefore not annotate our Manhattan plots with their results. Nevertheless the topology of their Manhattan plot for BTA 6 is strikingly similar to our results and results reported by [21]. When comparing plot C and D in more detail it becomes evident that combining iHS^Voight ^and WGA results does not give as good agreement between the combined iHS and WGA test. This is supported by the lower correlation among the top 1% iHS^Voight ^test statistics and regression coefficients from WGA (Table 3).

## Discussion

### Mixed model and method to control for stratification

The pairwise IBD matrix obtained by PLINK [60] based on genome wide SNP data most likely underestimated the relatedness among bulls because the underlying algorithm estimates population allele frequencies from a presumably unrelated sample. This is supported by the observation that the average IBD estimate was exactly 0.254 between 795 paternal half-sib pairs and not, as expected, elevated due to underlying distant relatedness. Stich et al. [61] used SPAGEDI software [62] to estimate the IBD matrix and noted a similar problem. SPAGEDI also assumes that random pairs of individuals are unrelated and assigns them a kinship coefficient of zero.

The „Q+K" method, proposed by [40], is a mixed model with Q, a matrix containing population substructure to estimate v, the vector of population effects and the kinship matrix K, which allows estimation of polygenic background effects based on information on familial relatedness from recent coancestry. The authors claimed improved control of the type I and type II error rates over other methods.

Applying method MIX instead of a least squares allelic regression substantially reduced the inflation factor λ from 2.02 to 1.34 for PY. When we extended method MIX by Q, the matrix on population substructure based on clusters, estimated using the „pairwise population concordance" criteria [40], *λ *was further reduced to 1.16 (data not shown) but still did not control for all of the stratification. The here proposed method MIXStrat was able to remove stratification (λ = 1.02) and proved an advantage over method „Q+K".

The Monte Carlo simulation confirms that the proposed MIXStrat approach deals correctly with all stratification in the data, as under the simulated H0 the observed -log *P *- Value distribution follows their expectation for the dataset as highly substructured as dairy cattle. If our two-step approach had resulted in an overcorrection we would expect to see deflation in the quantile - quantile plot.

### Detection of Selective Sweeps

Alleles under positive selection increase in frequency in a population and leave distinct signatures in the DNA sequence. One of these population-genetics based signatures is the increased length of the haplotype carrying the advantageous allele [6] which is caused by a rapid rise in frequency of the mutated allele. This creates temporary LD with nearby loci. Extended haplotype homozygosity statistics [6] contrast this signature between the ancestral and the derived allele at each locus.

The challenge is to determine whether a signature is due to selection or to confounding effects of population demographic history, such as bottlenecks, population expansions and population subdivision or simply due to drift in a finite population. Two striking bottlenecks were estimated by [63] in data from 14 European and African *Bos taurus *and *Bos indicus *cattle populations. The first and most prominent bottleneck occurred roughly 1,500 generations ago, which corresponds well with the time of domestication in cattle. The second less pronounced bottleneck, which occurred approximately 50 - 100 generations ago, is most likely caused by breed formation. We therefore expect substantial demographic noise in our set of selection signature test statistics. Furthermore consequent assortative mating is expected to leave signatures in the genome that can easily be mistaken as a signature of selection.

We mapped selection signatures with iHS^Voight^. Large negative values indicate regions in which newly derived alleles are increasing in frequency in the population. Large positive test statistics advocate so called soft sweeps, sweep from standing natural variation where the ancestral allele is increasing in frequency for iHS^Voight^. As changes in the selection regime of dairy cattle are well documented and make sweeps from standing genetic variation likely we believe that it is important to consider both extreme positive and negative iHS test statistics as potentially interesting regions in the cattle genome. We developed a permutation - based extension to the iHS statistic proposed by [9] for which there is no need to determine the ancestral and derived state of the alleles but contrasts minor and major allele. Our method obtains locus specific standard deviations of iHS in simulating the null hypothesis and contrasting against an expectation of zero. Compared to (iHS^Voight^) [9] our method is more conservative for loci with low minor allele frequencies. A higher correlation coefficient between our iHS and β_MIXStrat _indicates that this is a consequence of a decreased rate of false positive detections rather than reduced power. Despite successful selection signature scans in cattle we note that protein yield is a typical quantitative trait for which selection is essentially multigenic and therefore likely to undergo simultaneous selective sweeps. Chevin and Hospital [64] showed that for quantitative traits selection at specific quantitative trait loci may strongly vary in time and depend on the genetic background of the trait. This can blur the signature of selection and the corresponding region will go undetected in a genome scan [64]. Given the long generation intervals in cattle the number of generations of intense artificial selection is still small which could result in weak selection signals for alleles with small effects. Selection signature mapping applied to livestock with similarly strong selection but shorter generation intervals could be even more powerful.

### Method to combine Selection Signatures with Association signals

We propose a novel approach to increase the power to detect association signals. In this study the statistical power to detect an association signal was quite limited, but by combining two independent sources of information for QTL detection in genome wide studies: association and signatures of selection, we were able to increase power and to reduce the false positive rate. Loci that explain variation in economically important traits are likely under selection and will often show incomplete selective sweeps. Thus there is a good chance to observe extreme iHS values among loci that show association. This is supported by the positive correlation of 0.446 between β_MIXStrat _and iHS for loci among the top 1% iHS test statistics. Although many of the associations identified by our method are not yet confirmed, the concordance with prior results from WGA studies indicates that we were successful in detecting interesting loci. Fine mapping of QTL involves genotying of many more SNPs in the associated region possibly supported by resequencing a subset of extreme individuals [65] and is often tedious and costly. Thus it is highly desirable to eliminate false positive associations prior to further investigations.

Our combined approach has highest power at intermediate allele frequencies, as both independent sources of information (selection signature mapping and WGA) have highest power at intermediate allele frequencies. Alleles that are not allowed to go to fixation are either likely to be under balancing selection (heterozygote advantage) or have pleiotropic effects with positive and negative effects for the traits under selection. Such loci are not expected to show a signature of recent positive selection. WGA, given the same size of effect, will have equal power to identify such loci and loci under positive selection.

## Conclusion

The combination of WGA with hitchhiking mapping to identify a bona fide set of SNPs for candidate gene studies is very promising. We argue that our method improves power of QTL detection and reduces type I error rate by combining two independent sources of information. Our approach can of course be extended to all routinely recorded phenotypes, but for a proof of principle we restricted our analyses to PY as this trait was under most stringent selection over the last couple of decades and the bulls were selectively genotyped for PY to increase power for the whole genome association study.

Stratification is a substantial problem in WGA studies, particularly when carried out in livestock populations. Our MIXStrat approach controls the type I error rate, however at the cost of reduced power.

We accomplished a whole genome hitchhiking mapping study and identified roughly 1,600 SNPs displaying selection signatures that show generally good accordance with effects estimated in the WGA study. Our extension to the iHS test statistic proposed by [9] resulted in a reduced false positive rate in the MAF class < 10%, however, it provides reliable *P - *Values only after extensive Monte Carlo simulations.

Given the substantial increase in power and the reduction in false positive signals we recommend using our combined strategy rather than stand alone WGA. This is especially important in small populations where it is not possible to genotype additional animals.

## Competing interests

The authors declare that they have no competing interests.

## Authors' contributions

MD, HS, CS and RF, wrote the manuscript; KF, CW, FS produced genotyping data; HS, MD carried out the statistical analysis, HS, MD, CS and RF designed the study. All authors read and approved the final manuscript.

## Supplementary Material

Additional file 1 **Supplementary Figures S1-S5**. The PDF contains Figure S1: Histogram of means of 1000 permuted uIHS test statistics per locus; Figure S2: Histogram of derived allele frequencies for 34,851 SNPs in the study; Figure S3: Histogram of minor allele frequencies for 34,851 SNPs in the study; Figure S4: Histogram of *P *- Values of iHS^Voight ^test statistics; Figure S5: Histogram of *P *- Values of iHS test statistics. Click here for file

Additional file 2 **Supplementary Figures S6-S33**. The PDF shows Manhattan plots of bovine autosomes 1-5, 7-29; Capital letters denote QTLs reported from whole genome association studies (WGA) in cattle QTLdb at animalgenome.org, summarized as QTL trait ontology classes: B.. meat traits, E... exterior traits, H.. health traits, M.. milk traits, P.. production traits, R.. reproduction traits; o annotates a top 5% iHSVoight test statistic as reported in by [Quanbari et al. (2011)] in windows of 500 kb in Brown Swiss, x in any of the other breeds investigated; Plot A: iHSVoight test statistics, blue line: threshold identifying the top 5%; B: iHS test statistics, blue line: threshold identifying the top 5%; C: combined iHS^Voight ^and WGA results with model MIXstrat, D: combined iHS and WGA results with model MIXstrat; blue line is a at 10% false discovery rate threshold. Click here for file

Additional file 3 **Supplementary Figures S34-S36**. The PDF shows Figure S34: Histogram of allele substitution effects from whole genome association study employing model MIXstrat in kilogram protein yield; Figure S35: Histogram of Stouffer's *P *- Values of combined model MIXstrat and iHS^Voight ^test statistics; Figure S36: Histogram of Stouffer's *P *- Values of combined model MIXstrat and iHS test statistics. Click here for file
