# Supplementary figures and images for: Combining evidence of selection with association analysis increases power to detect regions influencing complex traits in dairy cattle
Source: BMC Genomics. 2012 Jan 30;13:48. doi: 10.1186/1471-2164-13-48 (PMC3305582; doi:10.1186/1471-2164-13-48)

Figure S1

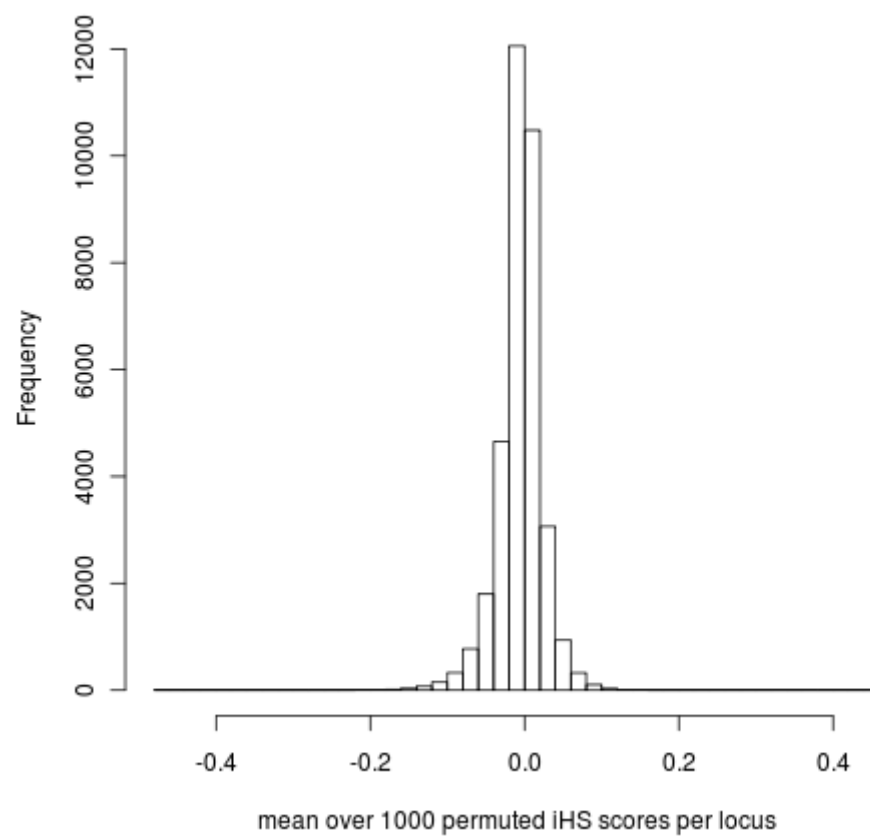

Figure S2

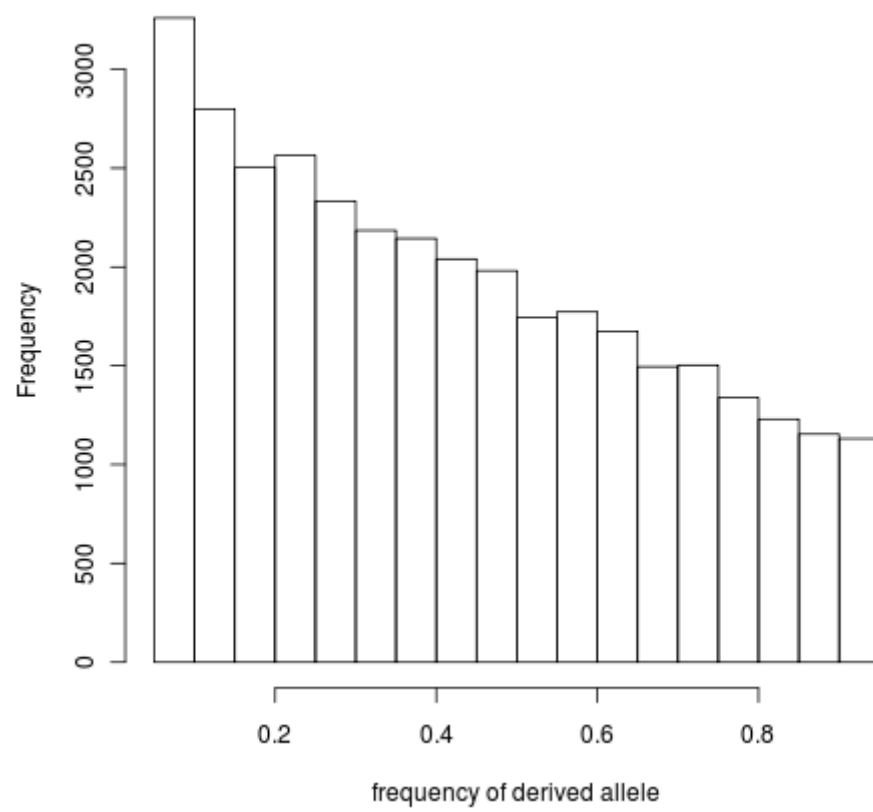

Figure S3:

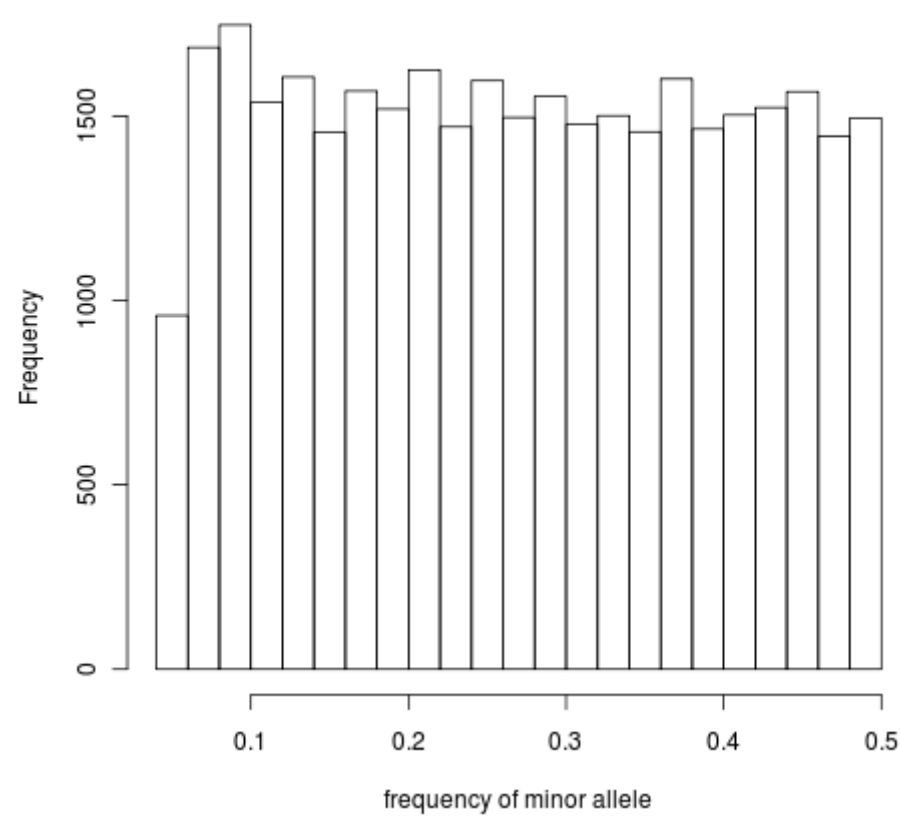

Figure S4

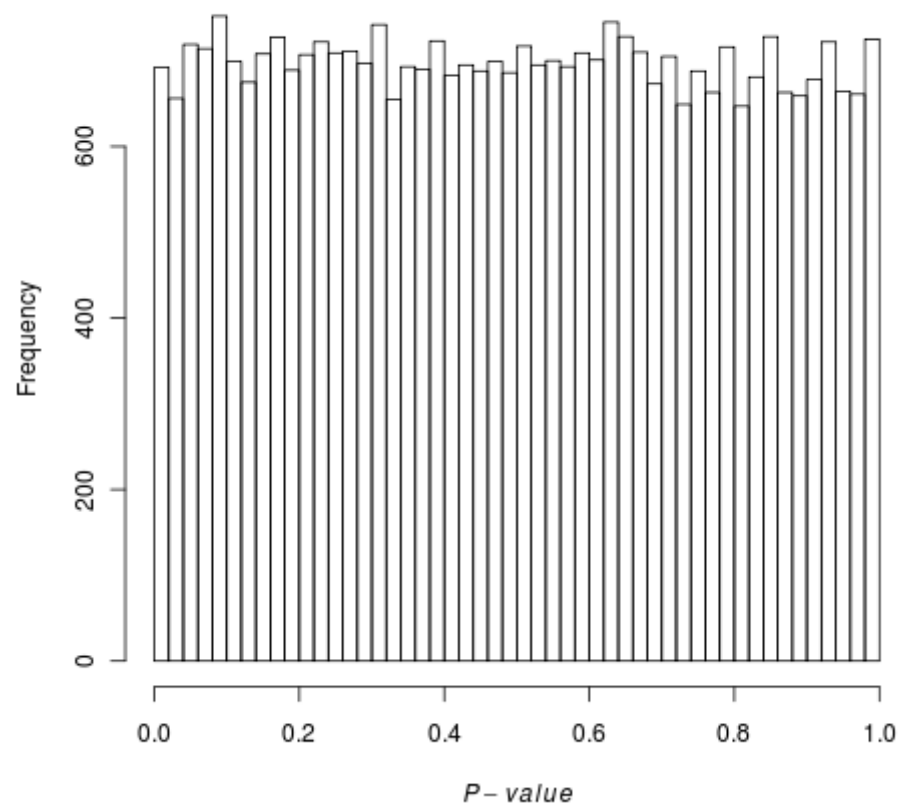

Figure S5

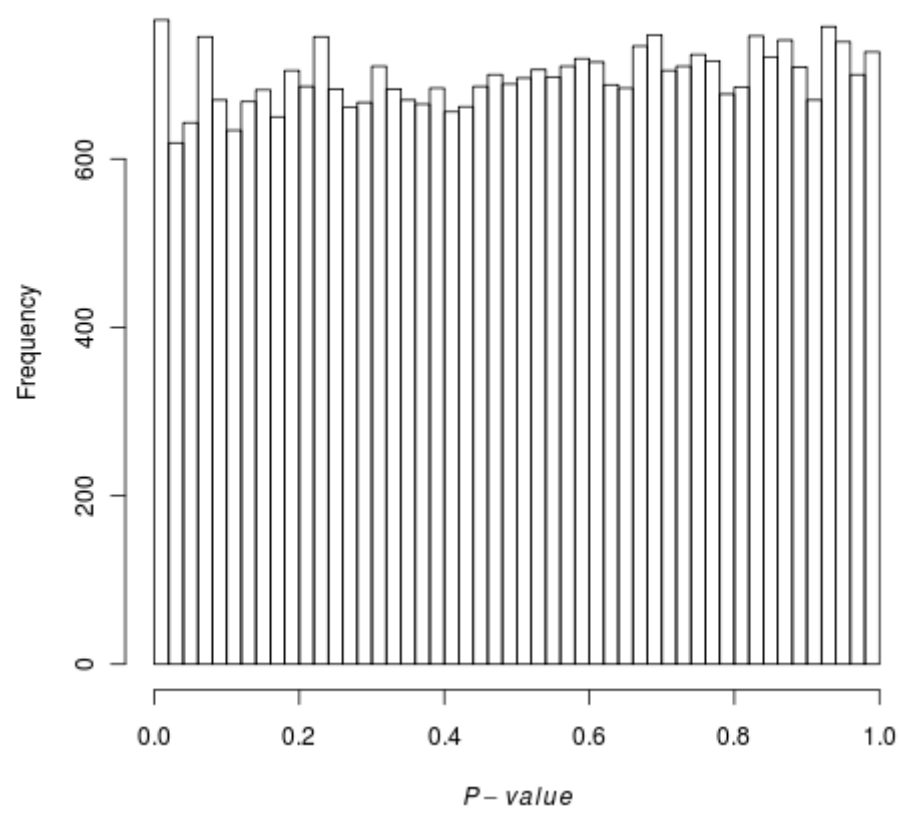

Supplement: Additional file 1 — Supplementary Figures S1-S5. The PDF contains Figure S1: Histogram of means of 1000 permuted uIHS test statistics per locus; Figure S2: Histogram of derived allele frequencies for 34,851 SNPs in the study; Figure S3: Histogram of minor allele frequencies for 34,851 SNPs in the study; Figure S4: Histogram of P - Values of iHSVoight test statistics; Figure S5: Histogram of P - Values of iHS test statistics. [file 1471-2164-13-48-S1.PDF]

Figure S34

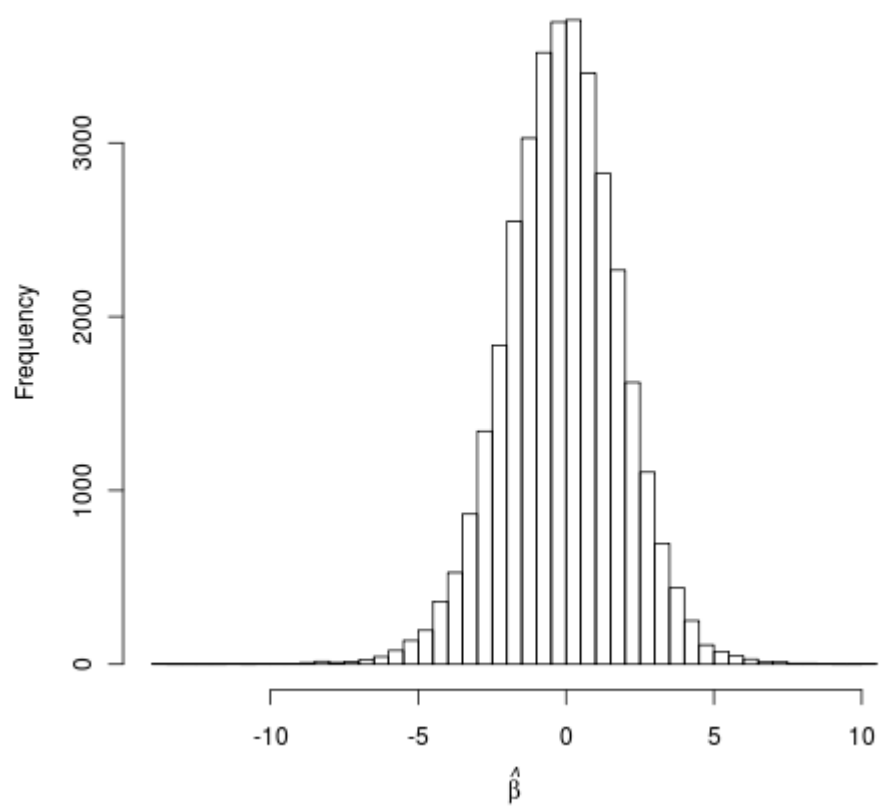

Figure S35

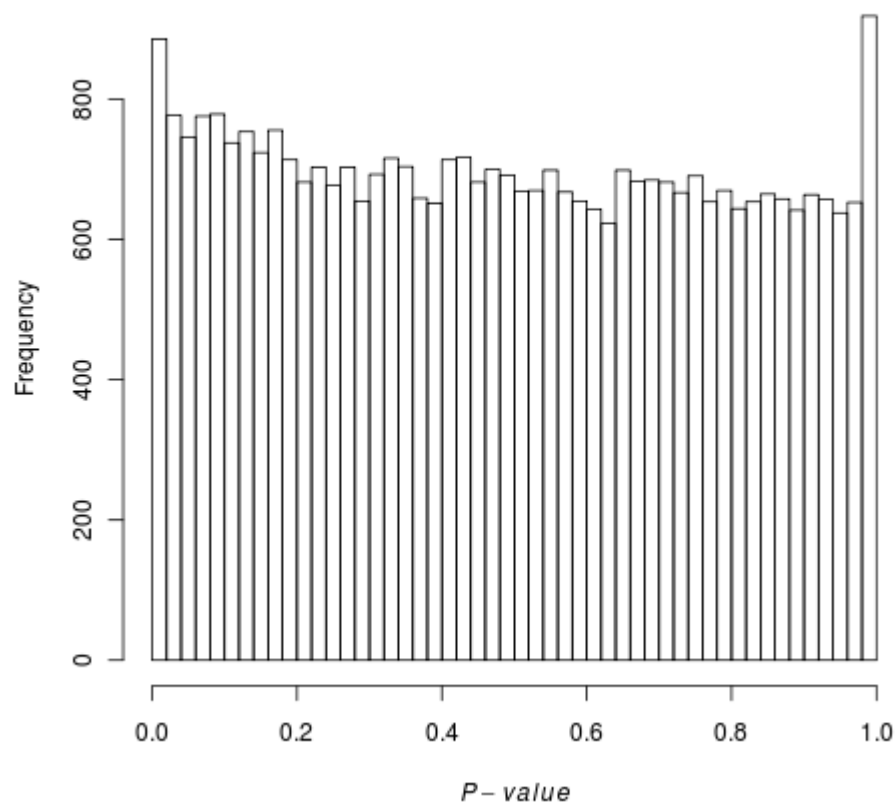

Figure S36

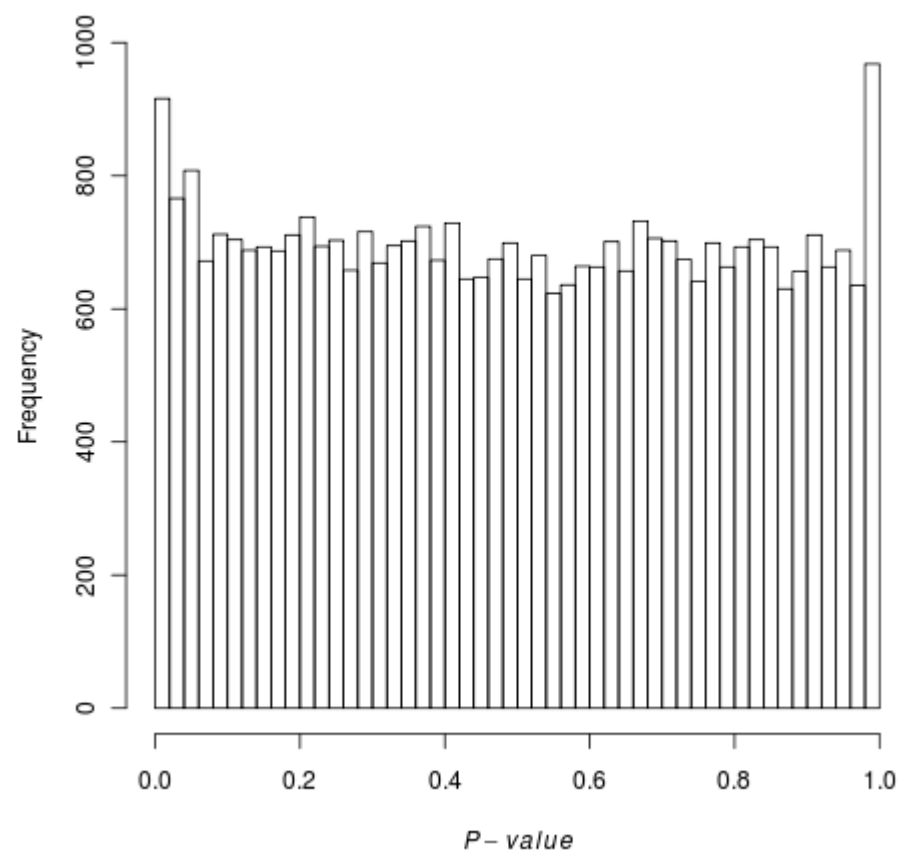

Supplement: Additional file 3 — Supplementary Figures S34-S36. The PDF shows Figure S34: Histogram of allele substitution effects from whole genome association study employing model MIXstrat in kilogram protein yield; Figure S35: Histogram of Stouffer's P - Values of combined model MIXstrat and iHSVoight test statistics; Figure S36: Histogram of Stouffer's P - Values of combined model MIXstrat and iHS test statistics. [file 1471-2164-13-48-S3.PDF]
