# Supplementary material for: Combining evidence of selection with association analysis increases power to detect regions influencing complex traits in dairy cattle
Source: BMC Genomics. 2012 Jan 30;13:48. doi: 10.1186/1471-2164-13-48 (PMC3305582; doi:10.1186/1471-2164-13-48)
Supplement: Additional file 2 — Supplementary Figures S6-S33. The PDF shows Manhattan plots of bovine autosomes 1-5, 7-29; Capital letters denote QTLs reported from whole genome association studies (WGA) in cattle QTLdb at animalgenome.org, summarized as QTL trait ontology classes: B.. meat traits, E... exterior traits, H.. health traits, M.. milk traits, P.. production traits, R.. reproduction traits; o annotates a top 5% iHSVoight test statistic as reported in by [Quanbari et al. (2011)] in windows of 500 kb in Brown Swiss, x in any of the other breeds investigated; Plot A: iHSVoight test statistics, blue line: threshold identifying the top 5%; B: iHS test statistics, blue line: threshold identifying the top 5%; C: combined iHSVoight and WGA results with model MIXstrat, D: combined iHS and WGA results with model MIXstrat; blue line is a at 10% false discovery rate threshold. [file 1471-2164-13-48-S2.PDF]

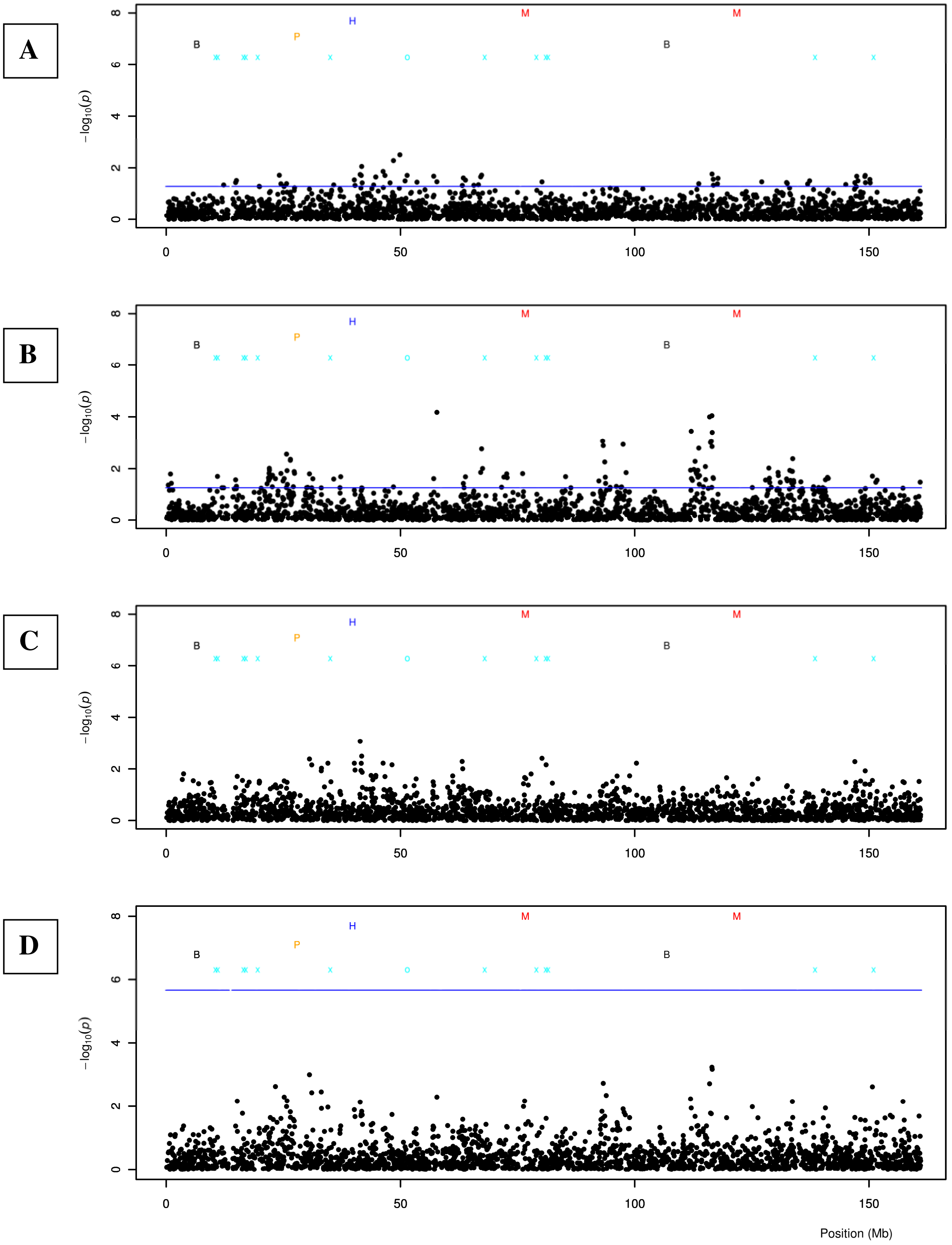

Figure S6: Manhattan plots of Chromosome 1; Capital letters denote QTLs reported from whole genome association studies (WGA) [52], summarized as QTL trait ontology classes: B.. meat traits, E... exterior traits, H.. health traits, M.. milk traits, P.. production traits, R.. reproduction traits; o annotates a top 5% $\text{iHS}^{\text{Voight}}$ test statistic as reported in by [18] in windows of 500 kb in Brown Swiss, x in any of the other breeds investigated; Plot A: $\text{iHS}^{\text{Voight}}$ test statistics, blue line: threshold identifying the top 5%; B: iHS test statistics, blue line: threshold identifying the top 5%; C: combined $\text{iHS}^{\text{Voight}}$ and WGA results with model MIXstrat, D: combined iHS and WGA result with model MIXstrat; blue line is a at 10% false discovery rate threshold

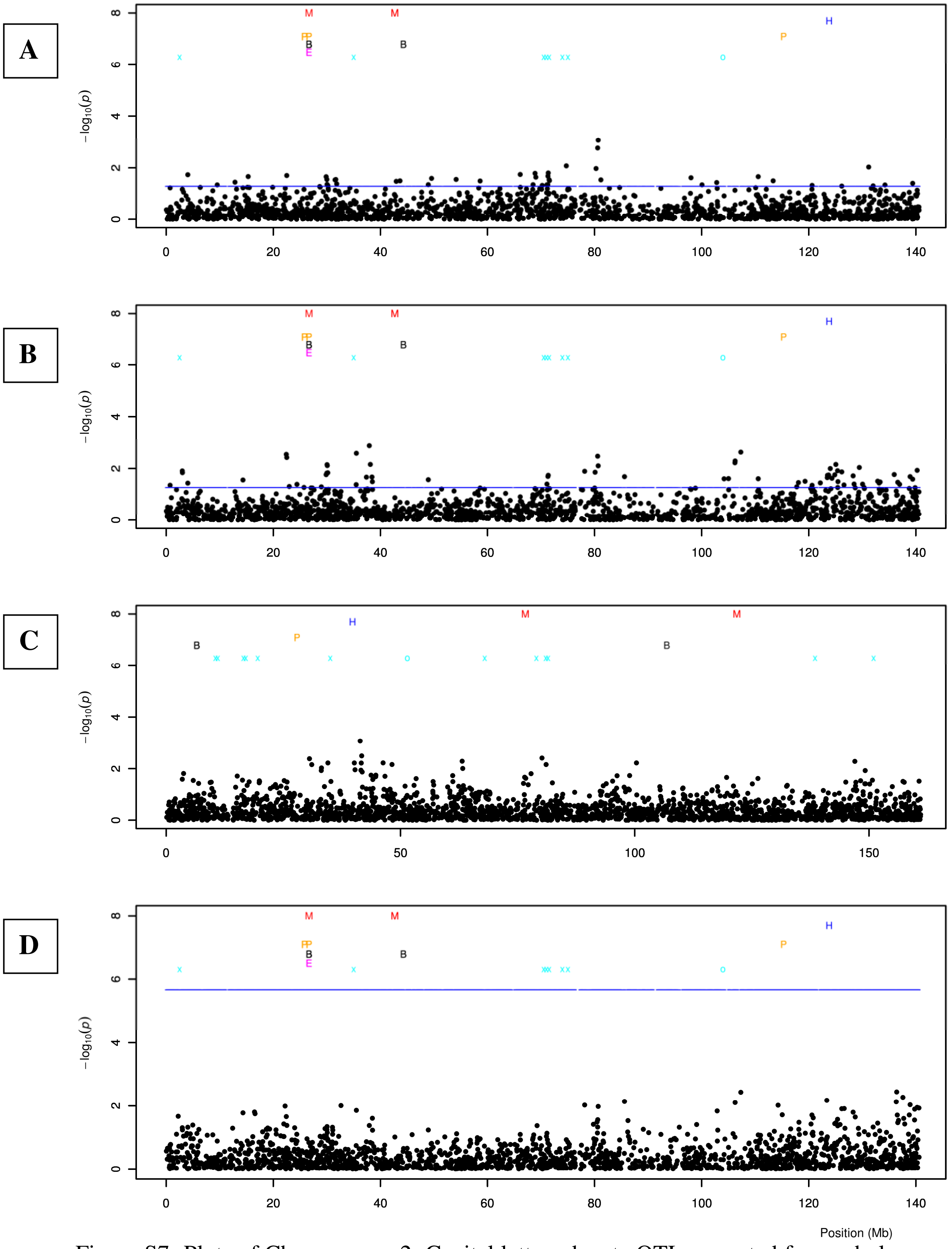

Figure S7: Plots of Chromosome 2; Capital letters denote QTLs reported from whole genome association studies (WGA) [52], summarized as QTL trait ontology classes: B.. meat traits, E... exterior traits, H.. health traits, M.. milk traits, P.. production traits, R.. reproduction traits; o annotates a top 5% iHS^{Voight} test statistic as reported in by [18] in windows of 500 kb in Brown Swiss, x in any of the other breeds investigated; Plot A: iHS^{Voight} test statistics, blue line: threshold identifying the top 5%; B: iHS test statistics, blue line: threshold identifying the top 5%; C: combined iHS^{Voight} and WGA results with model MIXstrat, D: combined iHS and WGA result with model MIXstrat; blue line is a at 10% false discovery rate threshold

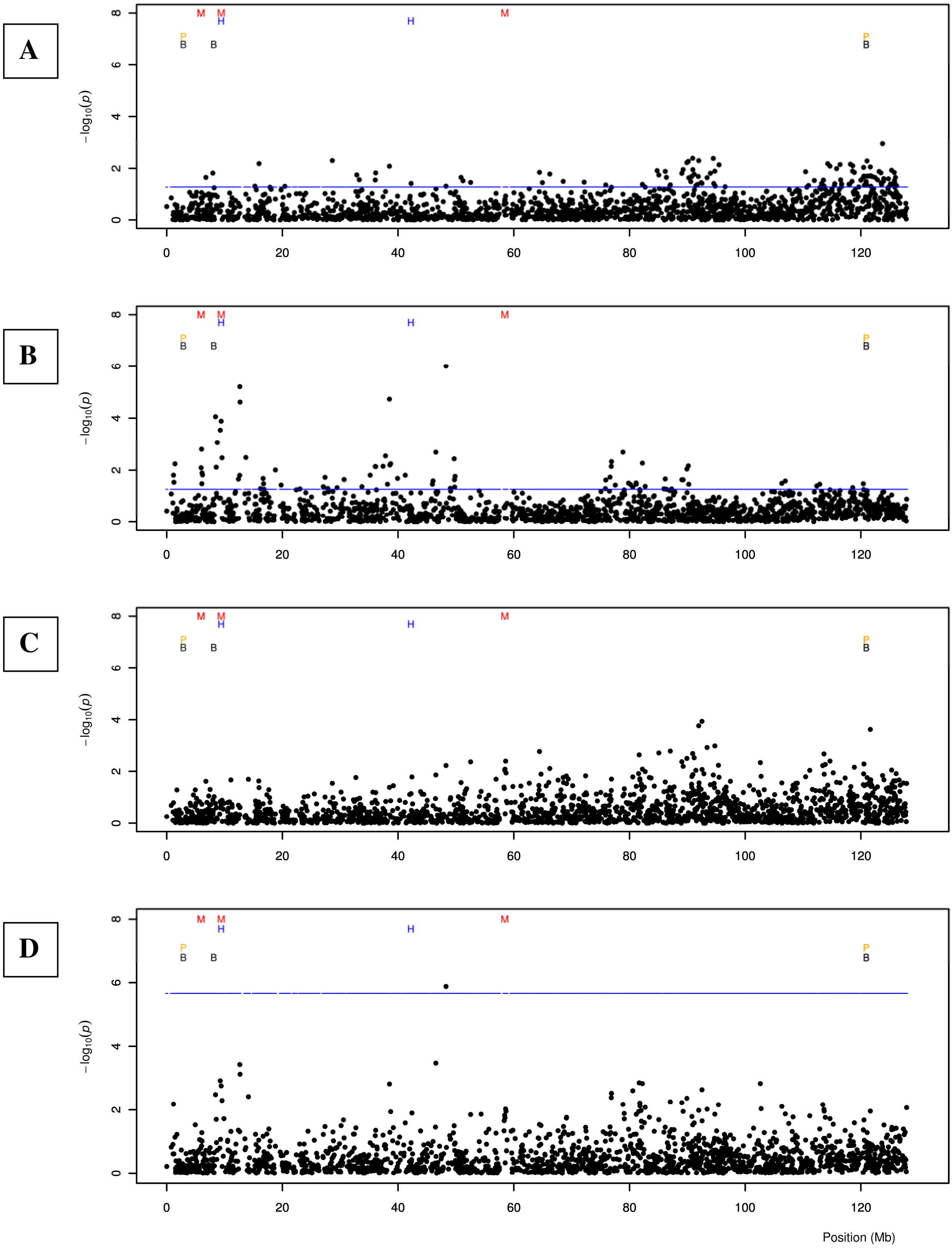

Figure S8: Plots of Chromosome 3; Capital letters denote QTLs reported from whole genome association studies (WGA) [52], summarized as QTL trait ontology classes: B.. meat traits, E... exterior traits, H.. health traits, M.. milk traits, P.. production traits, R.. reproduction traits; o annotates a top 5% iHS^{Voight} test statistic as reported in by [18] in windows of 500 kb in Brown Swiss, x in any of the other breeds investigated; Plot A: iHS^{Voight} test statistics, blue line: threshold identifying the top 5%; B: iHS test statistics, blue line: threshold identifying the top 5%; C: combined iHS^{Voight} and WGA results with model MIXstrat, D: combined iHS and WGA result with model MIXstrat; blue line is a at 10% false discovery rate threshold

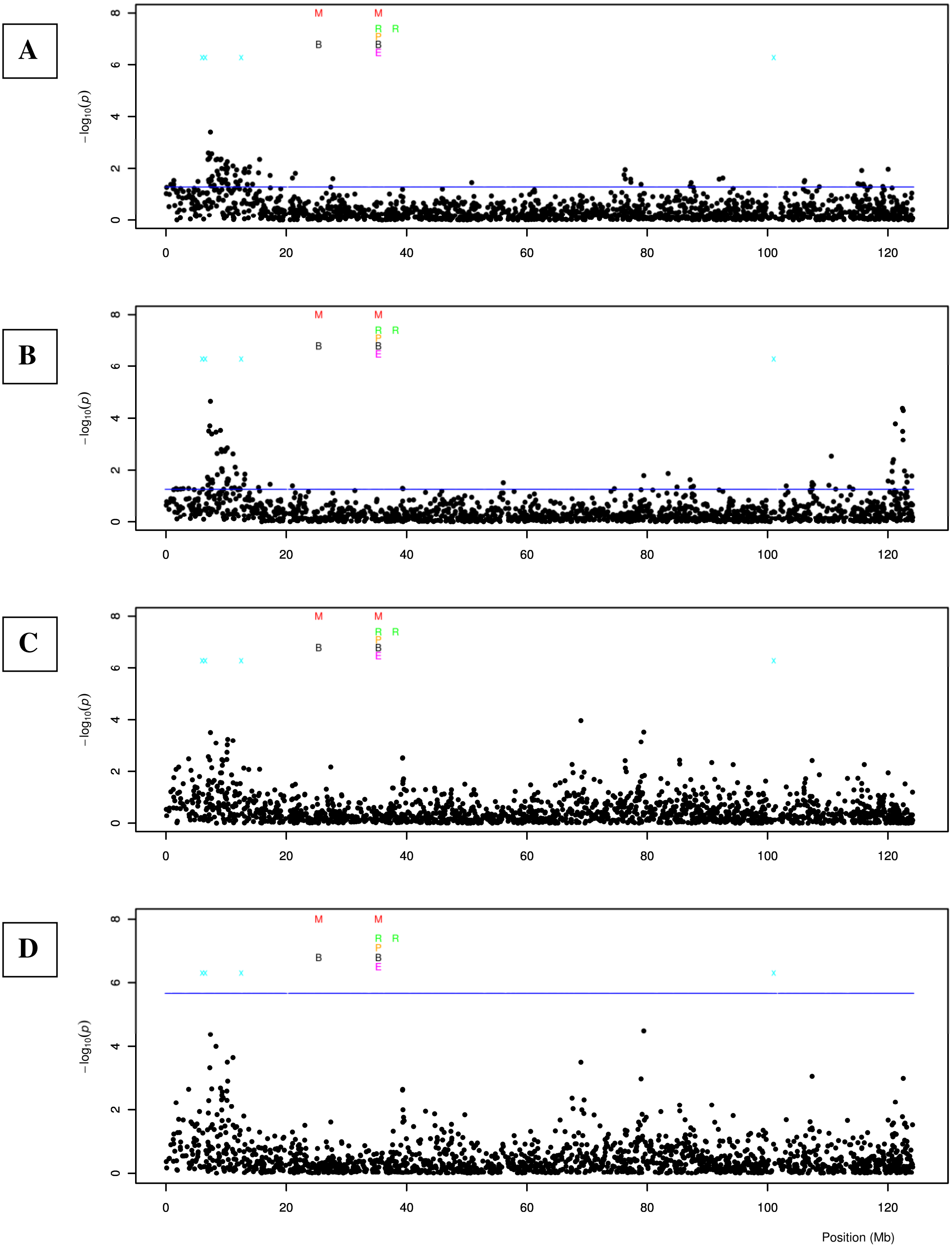

Figure S9: Plots of Chromosome 4; Capital letters denote QTLs reported from whole genome association studies (WGA) [52], summarized as QTL trait ontology classes: B.. meat traits, E... exterior traits, H.. health traits, M.. milk traits, P.. production traits, R.. reproduction traits; o annotates a top 5% iHS^{Voight} test statistic as reported in by [18] in windows of 500 kb in Brown Swiss, x in any of the other breeds investigated; Plot A: iHS^{Voight} test statistics, blue line: threshold identifying the top 5%; B: iHS test statistics, blue line: threshold identifying the top 5%; C: combined iHS^{Voight} and WGA results with model MIXstrat, D: combined iHS and WGA result with model MIXstrat; blue line is a at 10% false discovery rate threshold

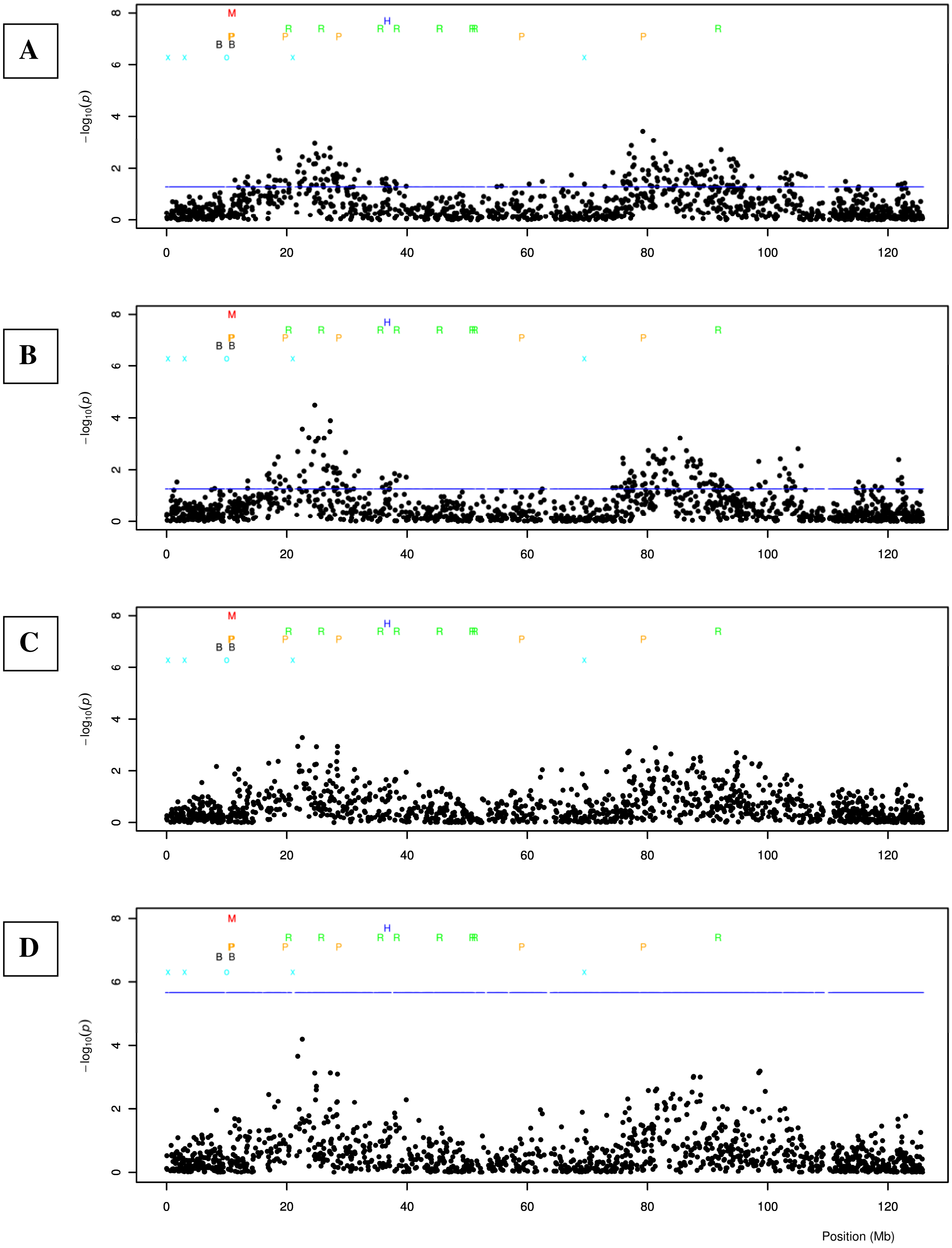

Figure S10: Plots of Chromosome 5; Capital letters denote QTLs reported from whole genome association studies (WGA) [52], summarized as QTL trait ontology classes: B.. meat traits, E... exterior traits, H.. health traits, M.. milk traits, P.. production traits, R.. reproduction traits; o annotates a top 5% $\text{iHS}^{\text{Voight}}$ test statistic as reported in by [18] in windows of 500 kb in Brown Swiss, x in any of the other breeds investigated; Plot A: $\text{iHS}^{\text{Voight}}$ test statistics, blue line: threshold identifying the top 5%; B: iHS test statistics, blue line: threshold identifying the top 5%; C: combined $\text{iHS}^{\text{Voight}}$ and WGA results with model MIXstrat, D: combined iHS and WGA result with model MIXstrat; blue line is a at 10% false discovery rate threshold

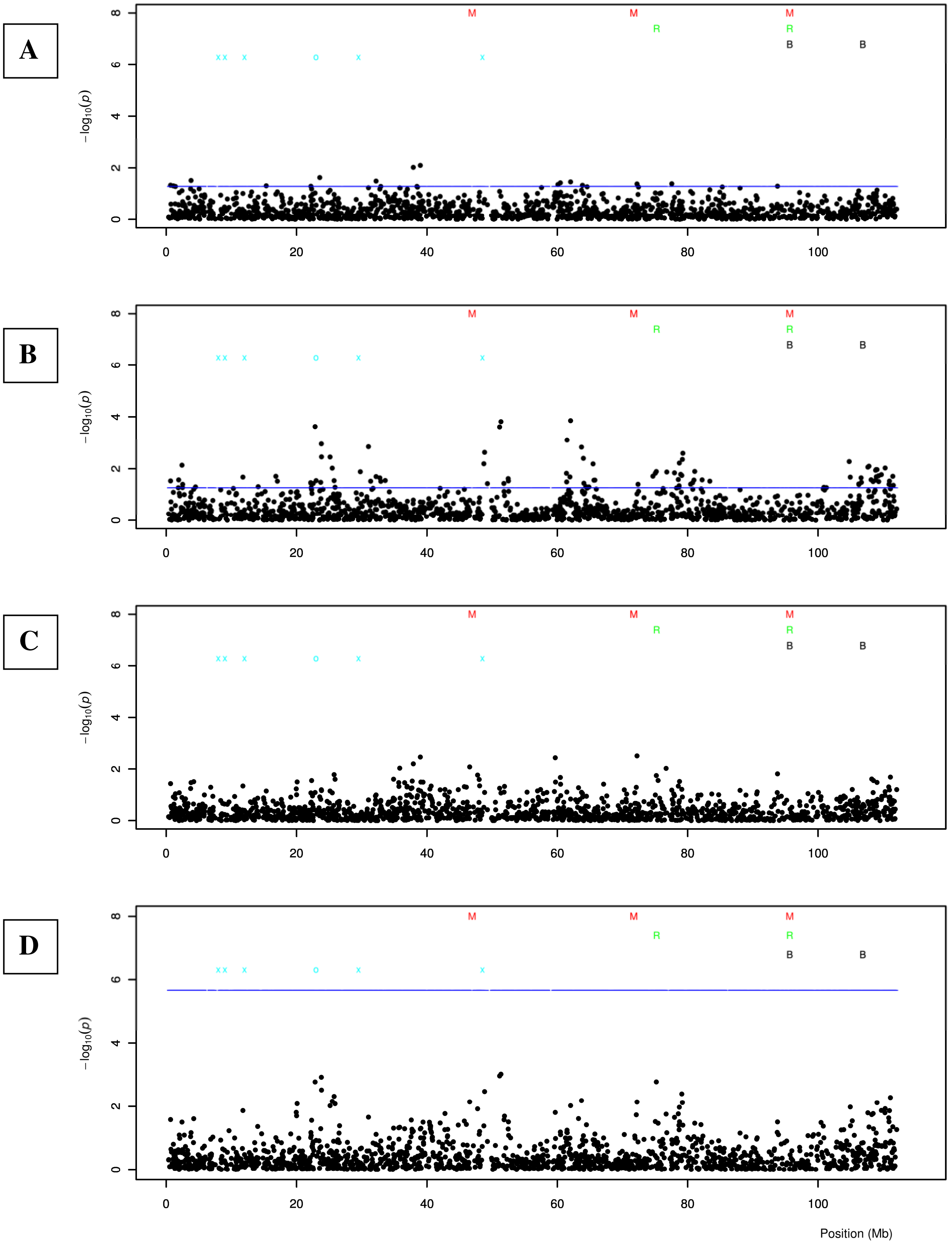

Figure S11: Plots of Chromosome 7; Capital letters denote QTLs reported from whole genome association studies (WGA) [52], summarized as QTL trait ontology classes: B.. meat traits, E... exterior traits, H.. health traits, M.. milk traits, P.. production traits, R.. reproduction traits; o annotates a top 5% iHS^{Voight} test statistic as reported in by [18] in windows of 500 kb in Brown Swiss, x in any of the other breeds investigated; Plot A: iHS^{Voight} test statistics, blue line: threshold identifying the top 5%; B: iHS test statistics, blue line: threshold identifying the top 5%; C: combined iHS^{Voight} and WGA results with model MIXstrat, D: combined iHS and WGA result with model MIXstrat; blue line is a at 10% false discovery rate threshold

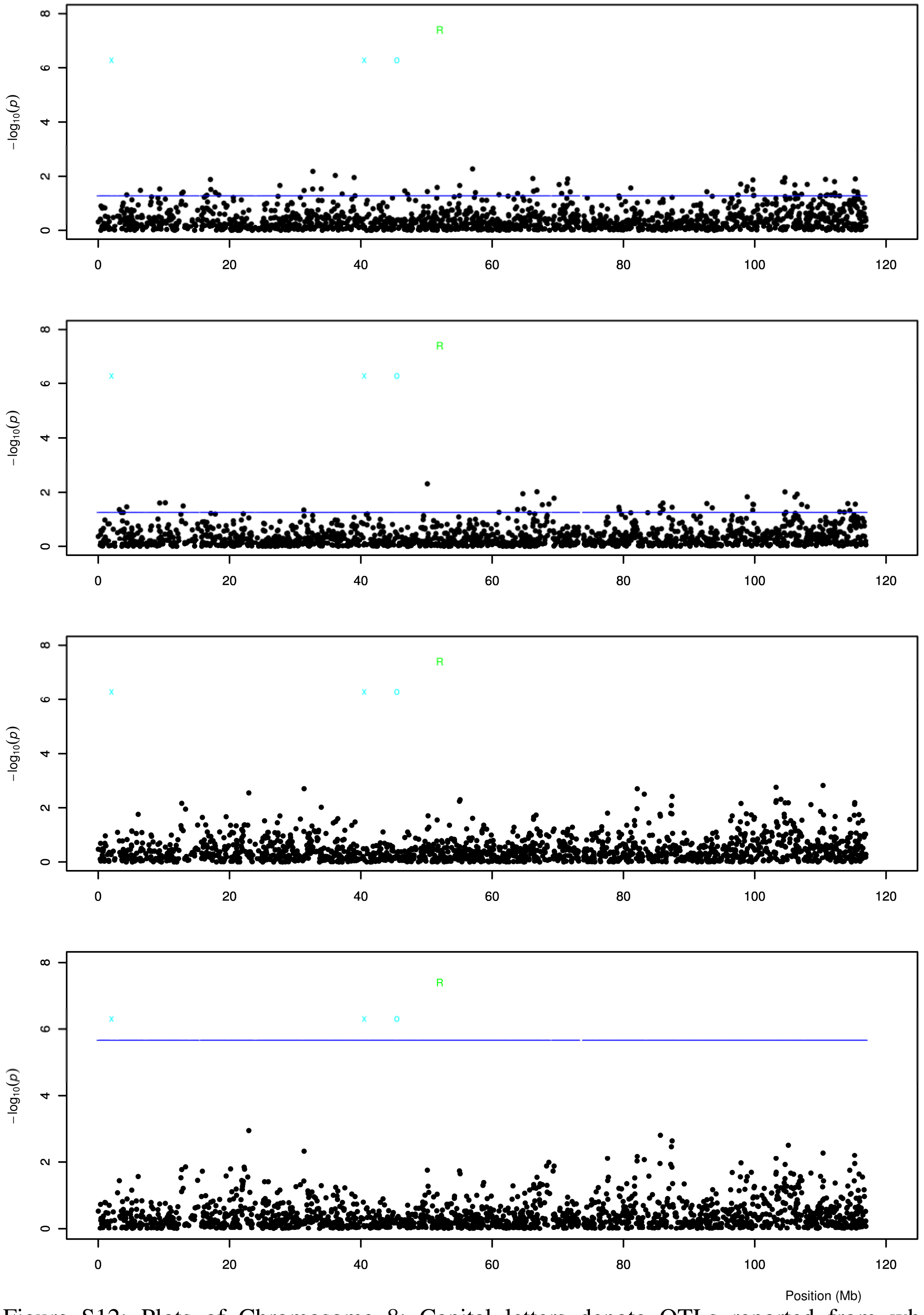

A

B

C

Figure S12: Plots of Chromosome 8; Capital letters denote QTLs reported from whole genome association studies (WGA) [52], summarized as QTL trait ontology classes: B.. meat traits, E... exterior traits, H.. health traits, M.. milk traits, P.. production traits, R.. reproduction traits; o annotates a top 5% iHS^{Voight} test statistic as reported in by [18] in windows of 500 kb in Brown Swiss, x in any of the other breeds investigated; Plot A: iHS^{Voight} test statistics, blue line: threshold identifying the top 5%; B: iHS test statistics, blue line: threshold identifying the top 5%; C: combined iHS^{Voight} and WGA results with model MIXstrat, D: combined iHS and WGA result with model MIXstrat; blue line is a at 10% false discovery rate threshold

D

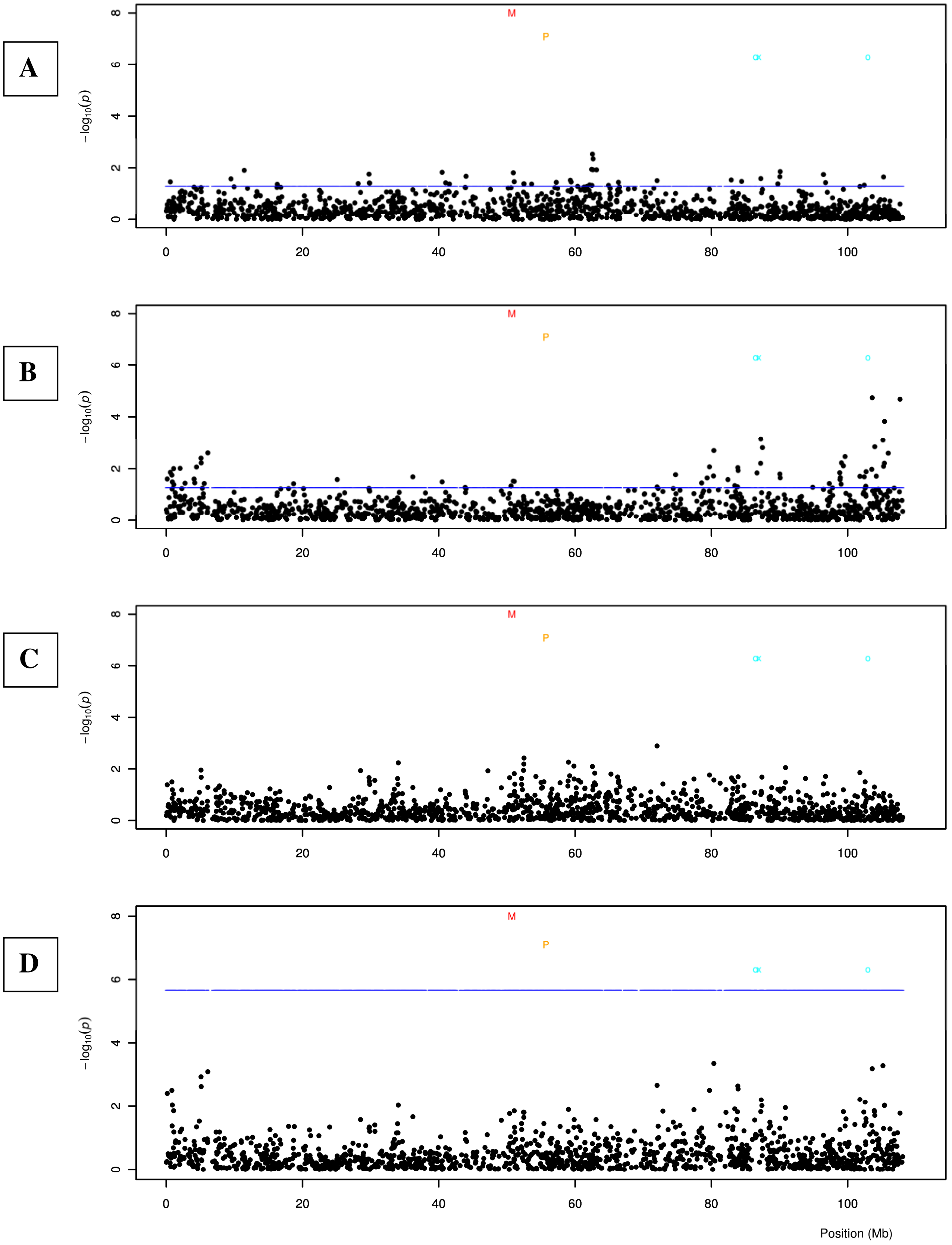

Figure S13: Plots of Chromosome 9; Capital letters denote QTLs reported from whole genome association studies (WGA) [52], summarized as QTL trait ontology classes: B.. meat traits, E... exterior traits, H.. health traits, M.. milk traits, P.. production traits, R.. reproduction traits; o annotates a top 5% iHS^{Voight} test statistic as reported in by [18] in windows of 500 kb in Brown Swiss, x in any of the other breeds investigated; Plot A: iHS^{Voight} test statistics, blue line: threshold identifying the top 5%; B: iHS test statistics, blue line: threshold identifying the top 5%; C: combined iHS^{Voight} and WGA results with model MIXstrat, D: combined iHS and WGA result with model MIXstrat; blue line is a at 10% false discovery rate threshold

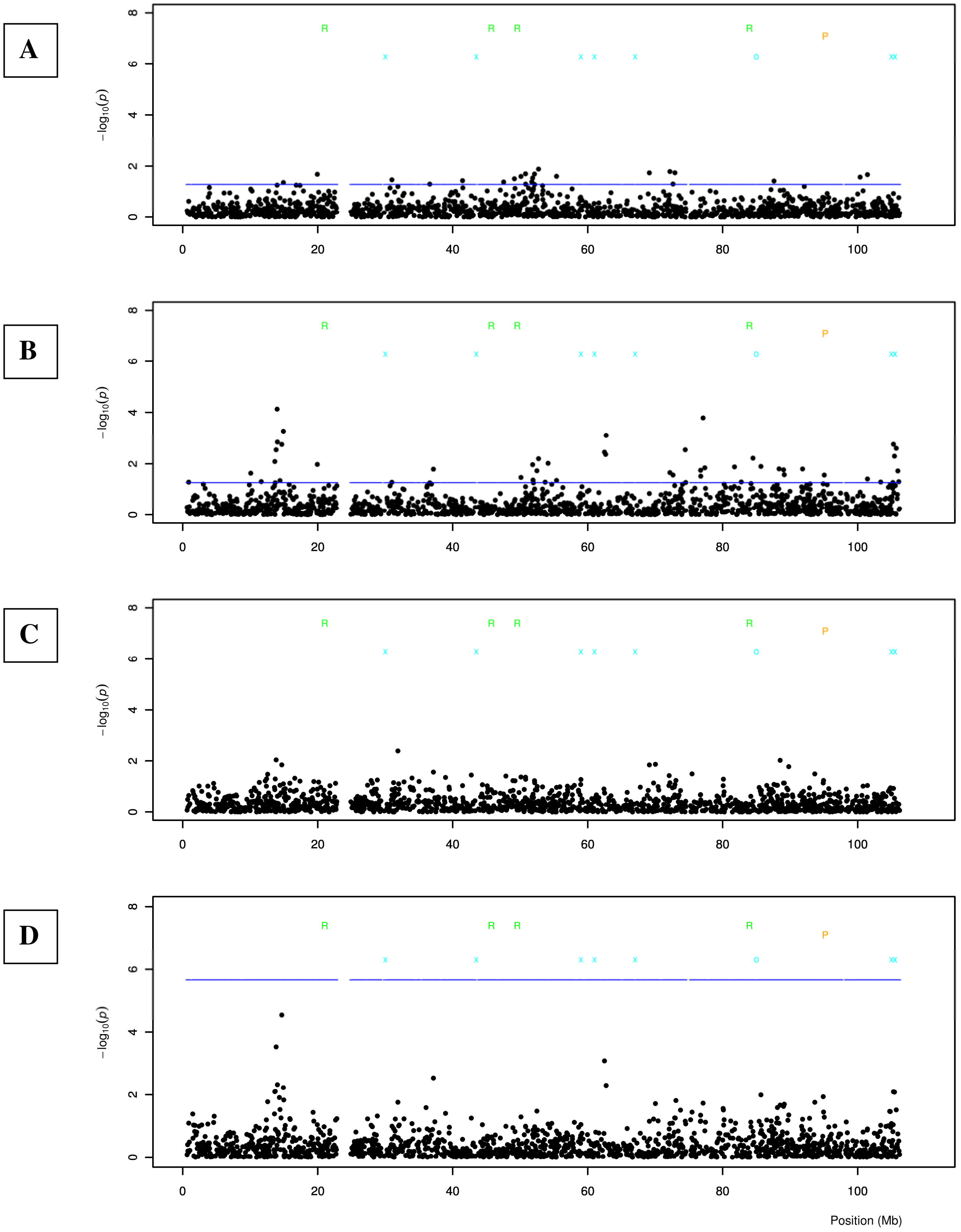

Figure S14: Plots of Chromosome 10; Capital letters denote QTLs reported from whole genome association studies (WGA) [52], summarized as QTL trait ontology classes: B.. meat traits, E... exterior traits, H.. health traits, M.. milk traits, P.. production traits, R.. reproduction traits; o annotates a top 5% iHS^{Voight} test statistic as reported in by [18] in windows of 500 kb in Brown Swiss, x in any of the other breeds investigated; Plot A: iHS^{Voight} test statistics, blue line: threshold identifying the top 5%; B: iHS test statistics, blue line: threshold identifying the top 5%; C: combined iHS^{Voight} and WGA results with model MIXstrat, D: combined iHS and WGA result with model MIXstrat; blue line is a at 10% false discovery rate threshold

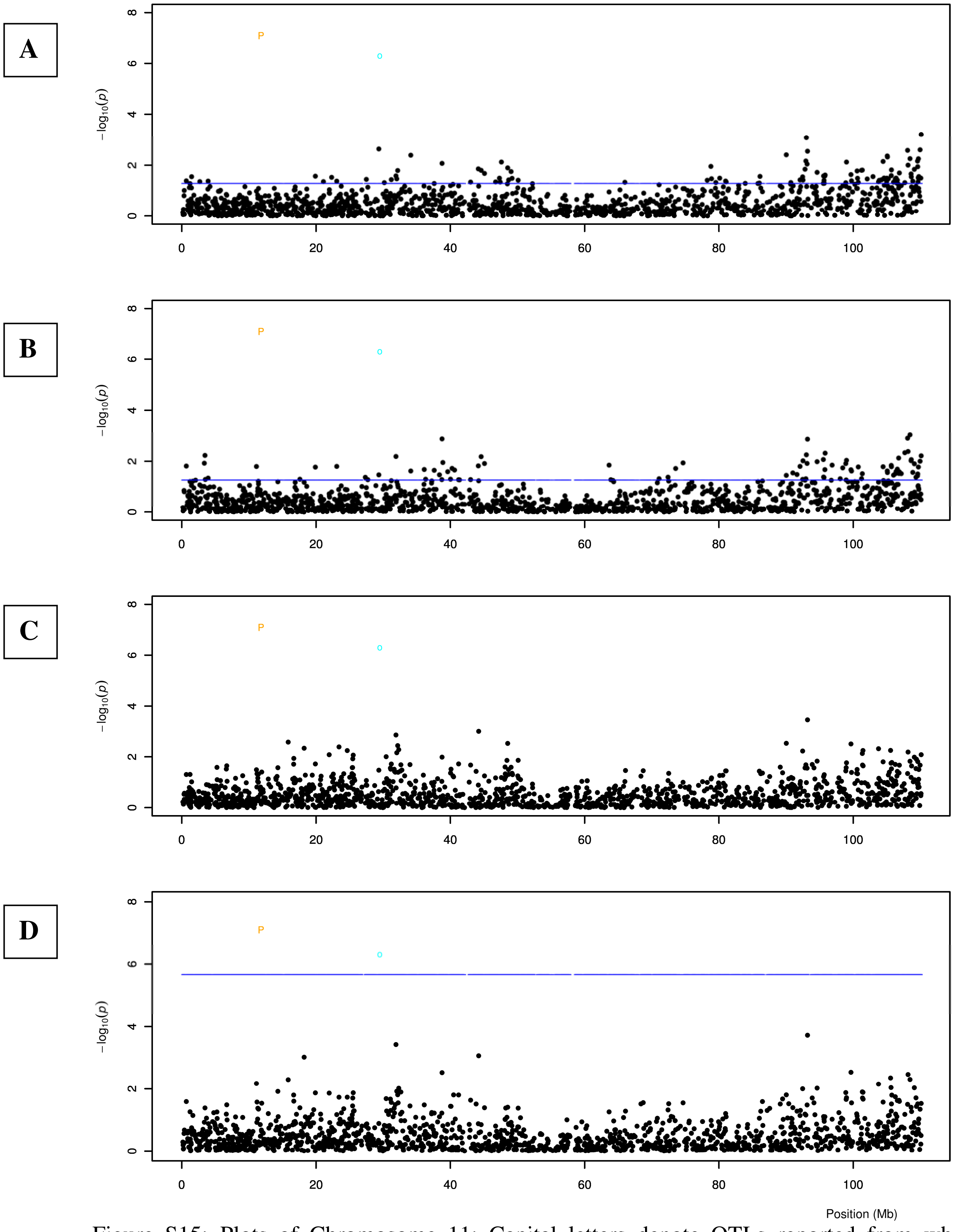

Figure S15: Plots of Chromosome 11; Capital letters denote QTLs reported from whole genome association studies (WGA) [52], summarized as QTL trait ontology classes: B.. meat traits, E... exterior traits, H.. health traits, M.. milk traits, P.. production traits, R.. reproduction traits; o annotates a top 5% iHS^{Voight} test statistic as reported in by [18] in windows of 500 kb in Brown Swiss, x in any of the other breeds investigated; Plot A: iHS^{Voight} test statistics, blue line: threshold identifying the top 5%; B: iHS test statistics, blue line: threshold identifying the top 5%; C: combined iHS^{Voight} and WGA results with model MIXstrat, D: combined iHS and WGA result with model MIXstrat; blue line is a at 10% false discovery rate threshold

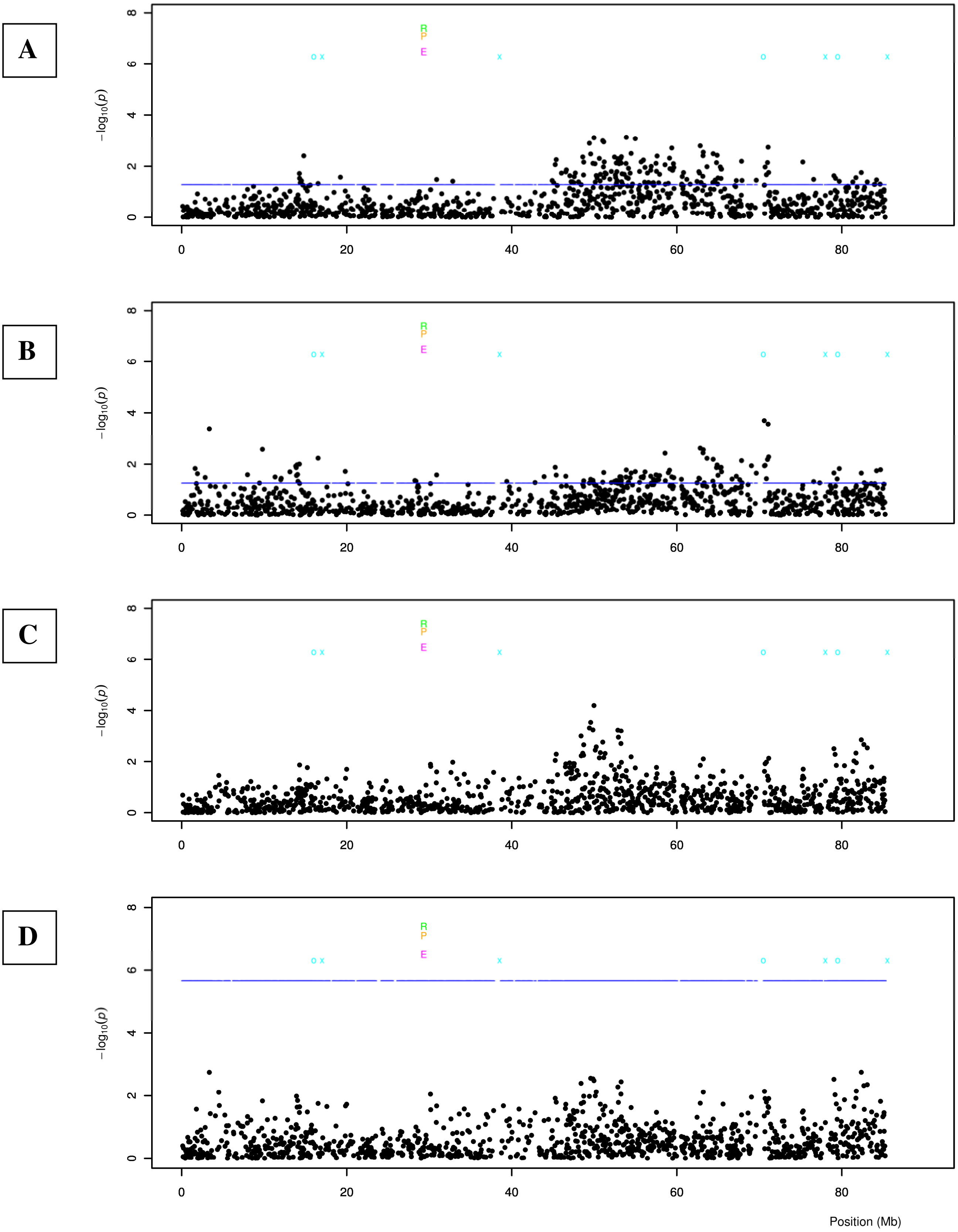

Figure S16: Plots of Chromosome 12; Capital letters denote QTLs reported from whole genome association studies (WGA) [52], summarized as QTL trait ontology classes: B.. meat traits, E... exterior traits, H.. health traits, M.. milk traits, P.. production traits, R.. reproduction traits; o annotates a top 5% $\text{iHS}^{\text{Voight}}$ test statistic as reported in by [18] in windows of 500 kb in Brown Swiss, x in any of the other breeds investigated; Plot A: $\text{iHS}^{\text{Voight}}$ test statistics, blue line: threshold identifying the top 5%; B: iHS test statistics, blue line: threshold identifying the top 5%; C: combined $\text{iHS}^{\text{Voight}}$ and WGA results with model MIXstrat, D: combined iHS and WGA result with model MIXstrat; blue line is a at 10% false discovery rate threshold

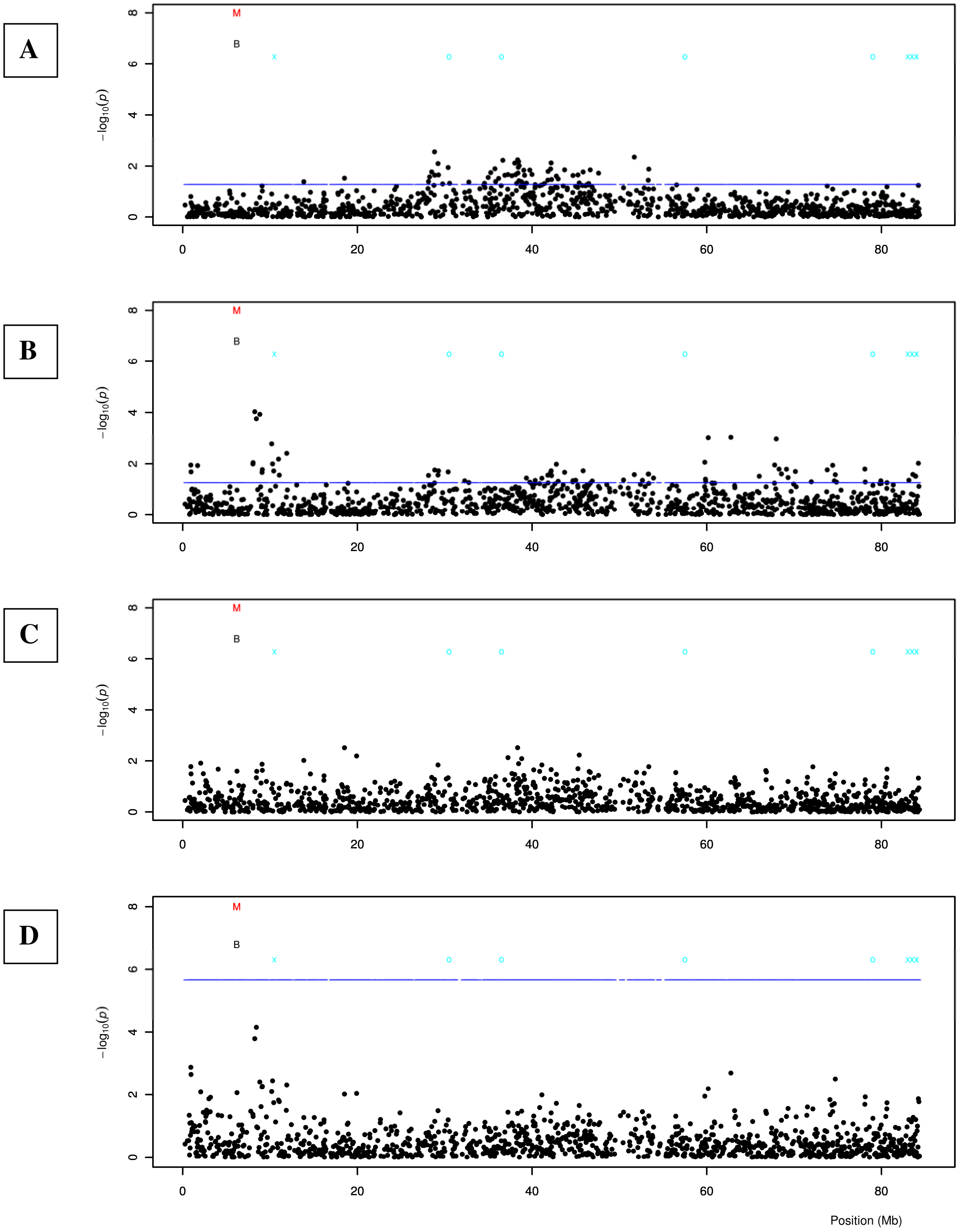

Figure S17: Plots of Chromosome 13; Capital letters denote QTLs reported from whole genome association studies (WGA) [52], summarized as QTL trait ontology classes: B.. meat traits, E... exterior traits, H.. health traits, M.. milk traits, P.. production traits, R.. reproduction traits; o annotates a top 5% iHS^{Voight} test statistic as reported in by [18] in windows of 500 kb in Brown Swiss, x in any of the other breeds investigated; Plot A: iHS^{Voight} test statistics, blue line: threshold identifying the top 5%; B: iHS test statistics, blue line: threshold identifying the top 5%; C: combined iHS^{Voight} and WGA results with model MIXstrat, D: combined iHS and WGA result with model MIXstrat; blue line is a at 10% false discovery rate threshold

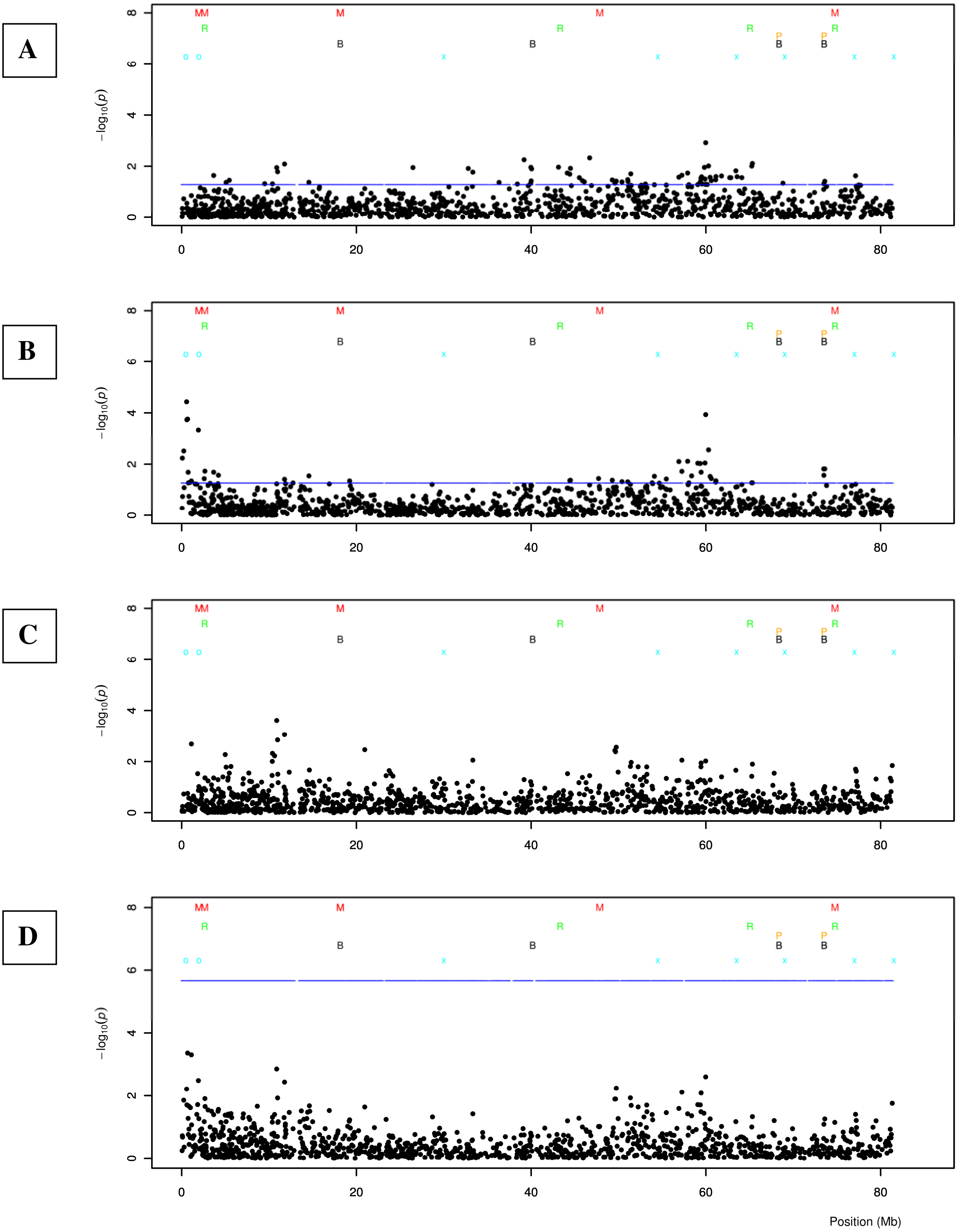

Figure S18: Plots of Chromosome 14; Capital letters denote QTLs reported from whole genome association studies (WGA) [52], summarized as QTL trait ontology classes: B.. meat traits, E... exterior traits, H.. health traits, M.. milk traits, P.. production traits, R.. reproduction traits; o annotates a top 5% iHS^{Voight} test statistic as reported in by [18] in windows of 500 kb in Brown Swiss, x in any of the other breeds investigated; Plot A: iHS^{Voight} test statistics, blue line: threshold identifying the top 5%; B: iHS test statistics, blue line: threshold identifying the top 5%; C: combined iHS^{Voight} and WGA results with model MIXstrat, D: combined iHS and WGA result with model MIXstrat; blue line is a at 10% false discovery rate threshold

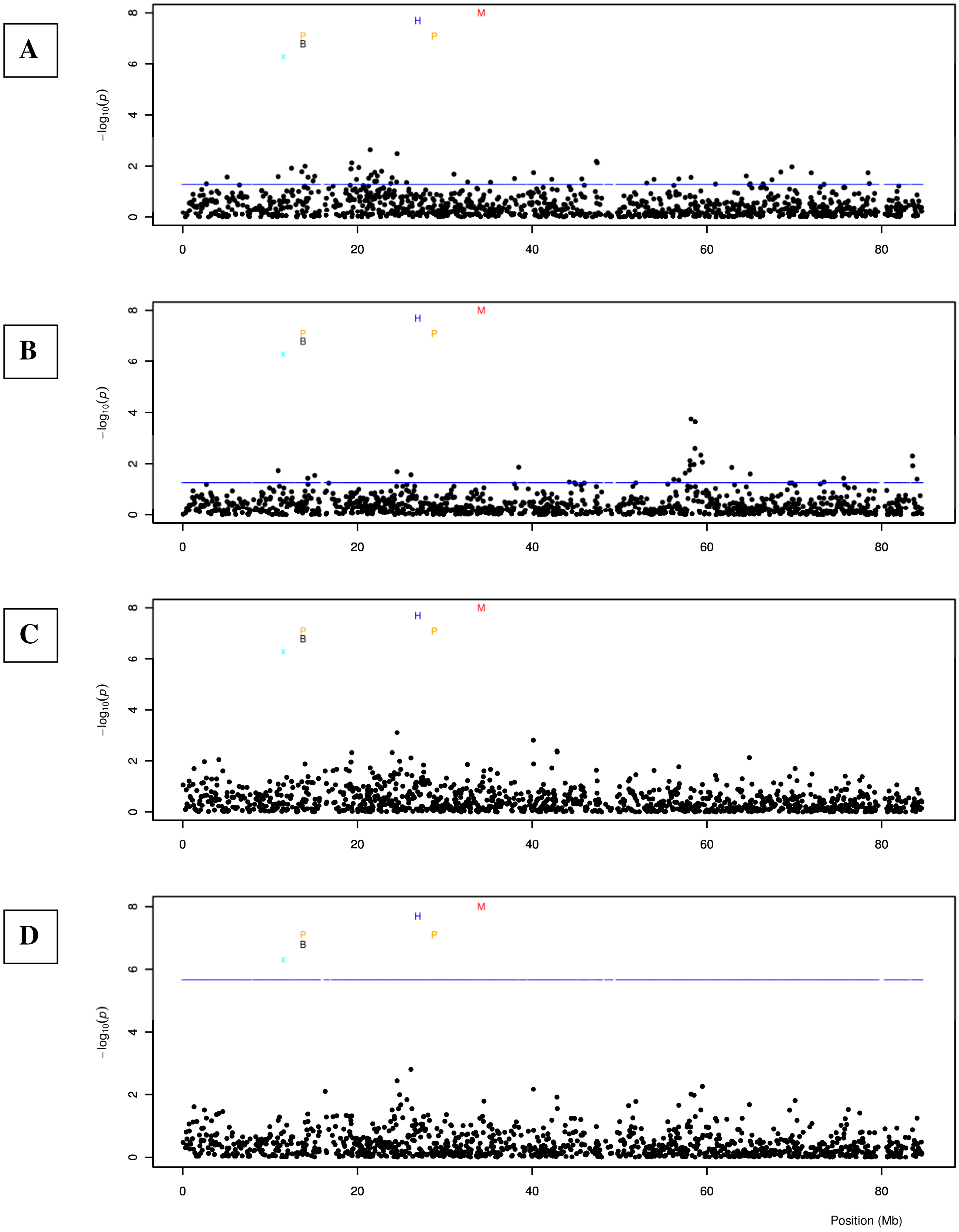

Figure S19: Plots of Chromosome 15; Capital letters denote QTLs reported from whole genome association studies (WGA) [52], summarized as QTL trait ontology classes: B.. meat traits, E... exterior traits, H.. health traits, M.. milk traits, P.. production traits, R.. reproduction traits; o annotates a top 5% $\text{iHS}^{\text{Voight}}$ test statistic as reported in by [18] in windows of 500 kb in Brown Swiss, x in any of the other breeds investigated; Plot A: $\text{iHS}^{\text{Voight}}$ test statistics, blue line: threshold identifying the top 5%; B: iHS test statistics, blue line: threshold identifying the top 5%; C: combined $\text{iHS}^{\text{Voight}}$ and WGA results with model MIXstrat, D: combined iHS and WGA result with model MIXstrat; blue line is a at 10% false discovery rate threshold

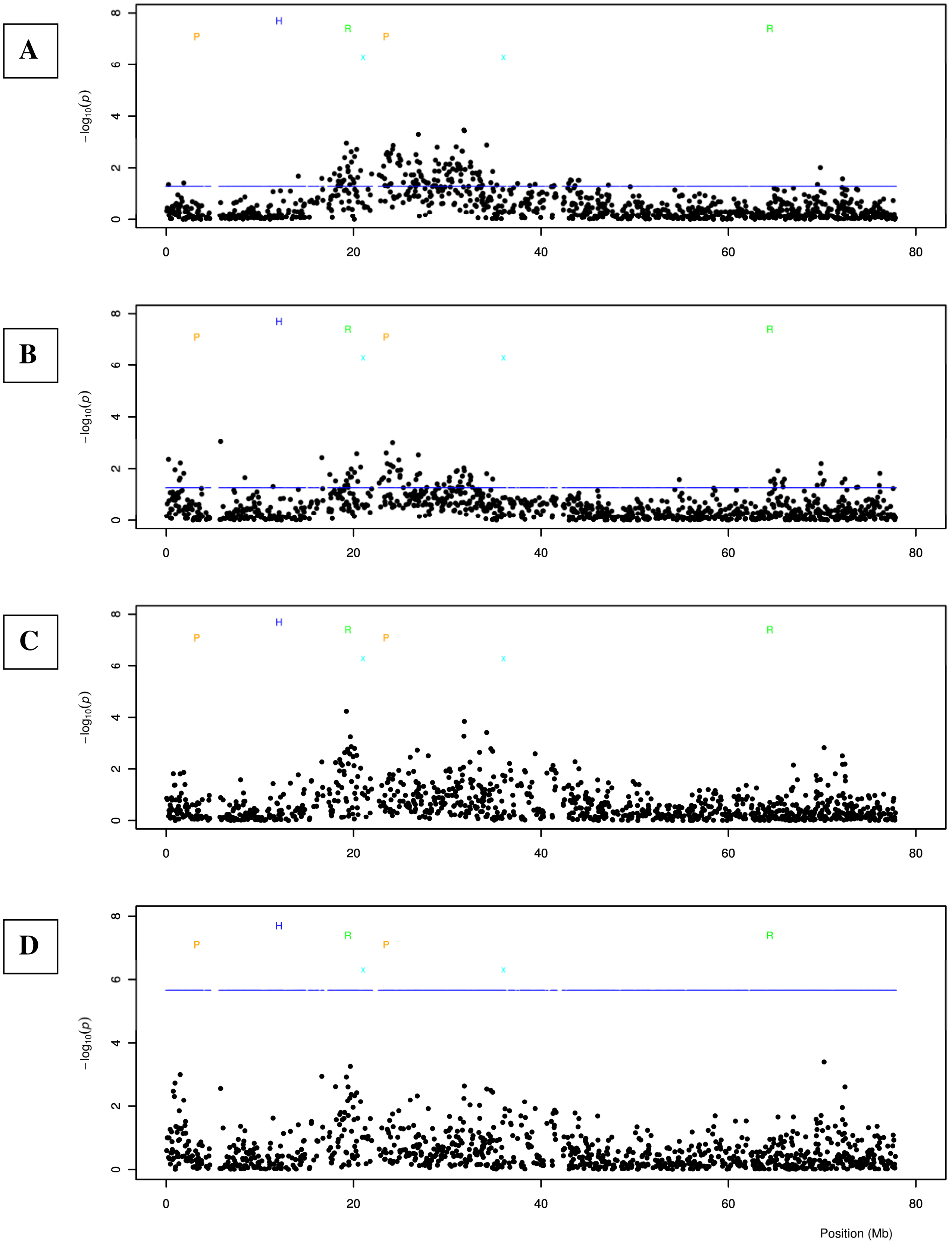

Figure S20: Plots of Chromosome 16; Capital letters denote QTLs reported from whole genome association studies (WGA) [52], summarized as QTL trait ontology classes: B.. meat traits, E... exterior traits, H.. health traits, M.. milk traits, P.. production traits, R.. reproduction traits; o annotates a top 5% iHS^{Voight} test statistic as reported in by [18] in windows of 500 kb in Brown Swiss, x in any of the other breeds investigated; Plot A: iHS^{Voight} test statistics, blue line: threshold identifying the top 5%; B: iHS test statistics, blue line: threshold identifying the top 5%; C: combined iHS^{Voight} and WGA results with model MIXstrat, D: combined iHS and WGA result with model MIXstrat; blue line is a at 10% false discovery rate threshold

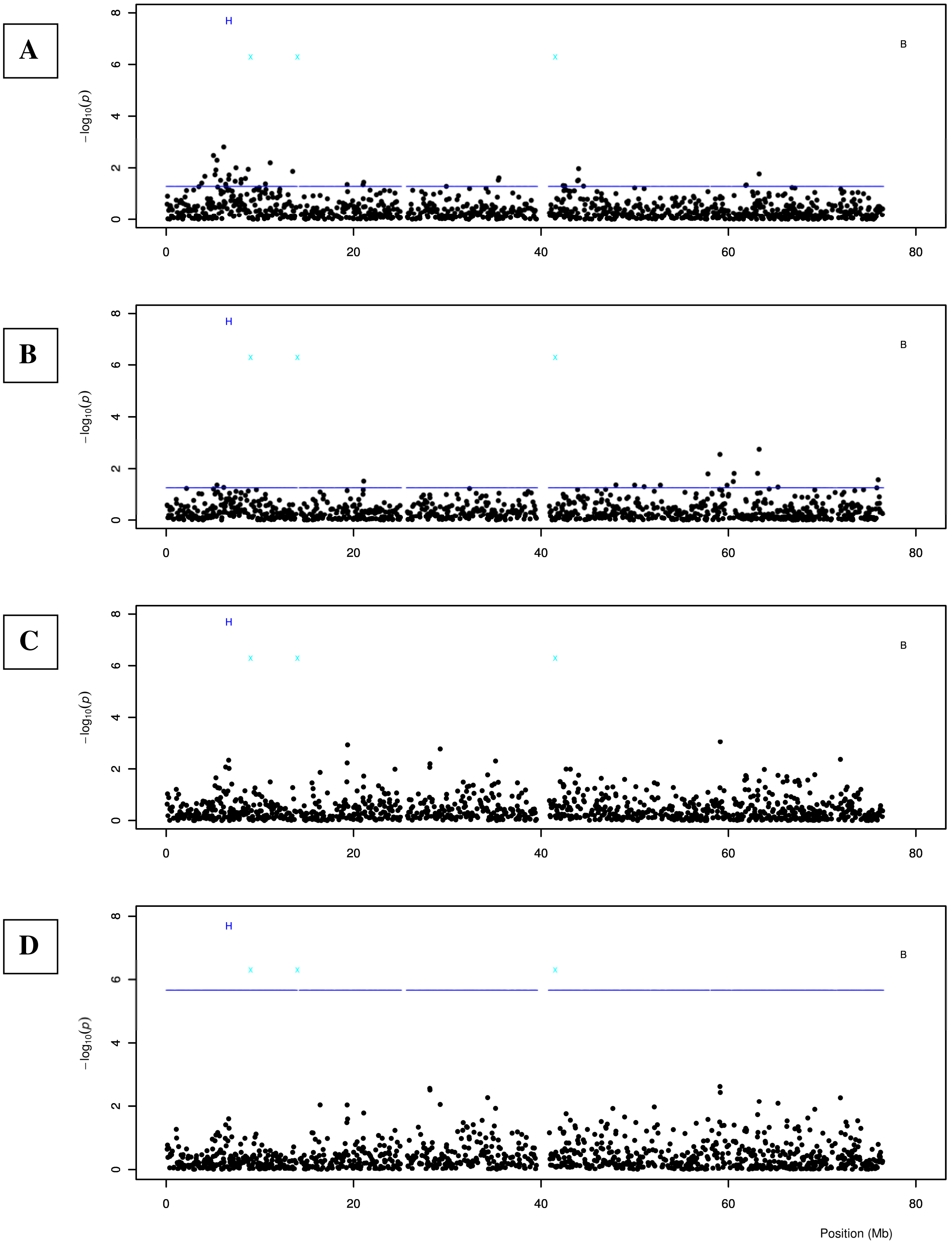

Figure S21: Plots of Chromosome 17; Capital letters denote QTLs reported from whole genome association studies (WGA) [52], summarized as QTL trait ontology classes: B.. meat traits, E... exterior traits, H.. health traits, M.. milk traits, P.. production traits, R.. reproduction traits; o annotates a top 5% iHS^{Voight} test statistic as reported in by [18] in windows of 500 kb in Brown Swiss, x in any of the other breeds investigated; Plot A: iHS^{Voight} test statistics, blue line: threshold identifying the top 5%; B: iHS test statistics, blue line: threshold identifying the top 5%; C: combined iHS^{Voight} and WGA results with model MIXstrat, D: combined iHS and WGA result with model MIXstrat; blue line is a at 10% false discovery rate threshold

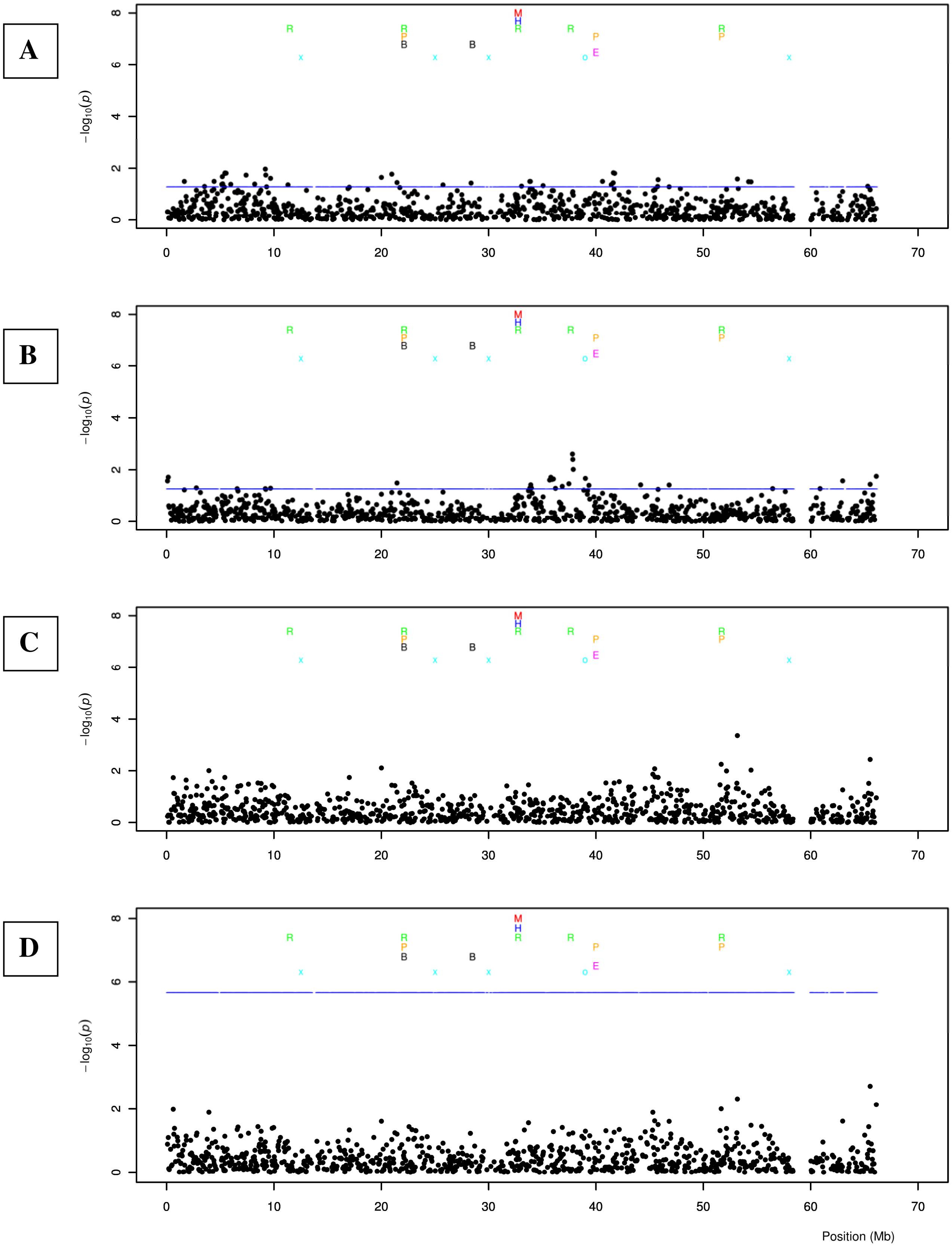

Figure S22: Plots of Chromosome 18; Capital letters denote QTLs reported from whole genome association studies (WGA) [52], summarized as QTL trait ontology classes: B.. meat traits, E... exterior traits, H.. health traits, M.. milk traits, P.. production traits, R.. reproduction traits; o annotates a top 5% iHS^{Voight} test statistic as reported in by [18] in windows of 500 kb in Brown Swiss, x in any of the other breeds investigated; Plot A: iHS^{Voight} test statistics, blue line: threshold identifying the top 5%; B: iHS test statistics, blue line: threshold identifying the top 5%; C: combined iHS^{Voight} and WGA results with model MIXstrat, D: combined iHS and WGA result with model MIXstrat; blue line is a at 10% false discovery rate threshold

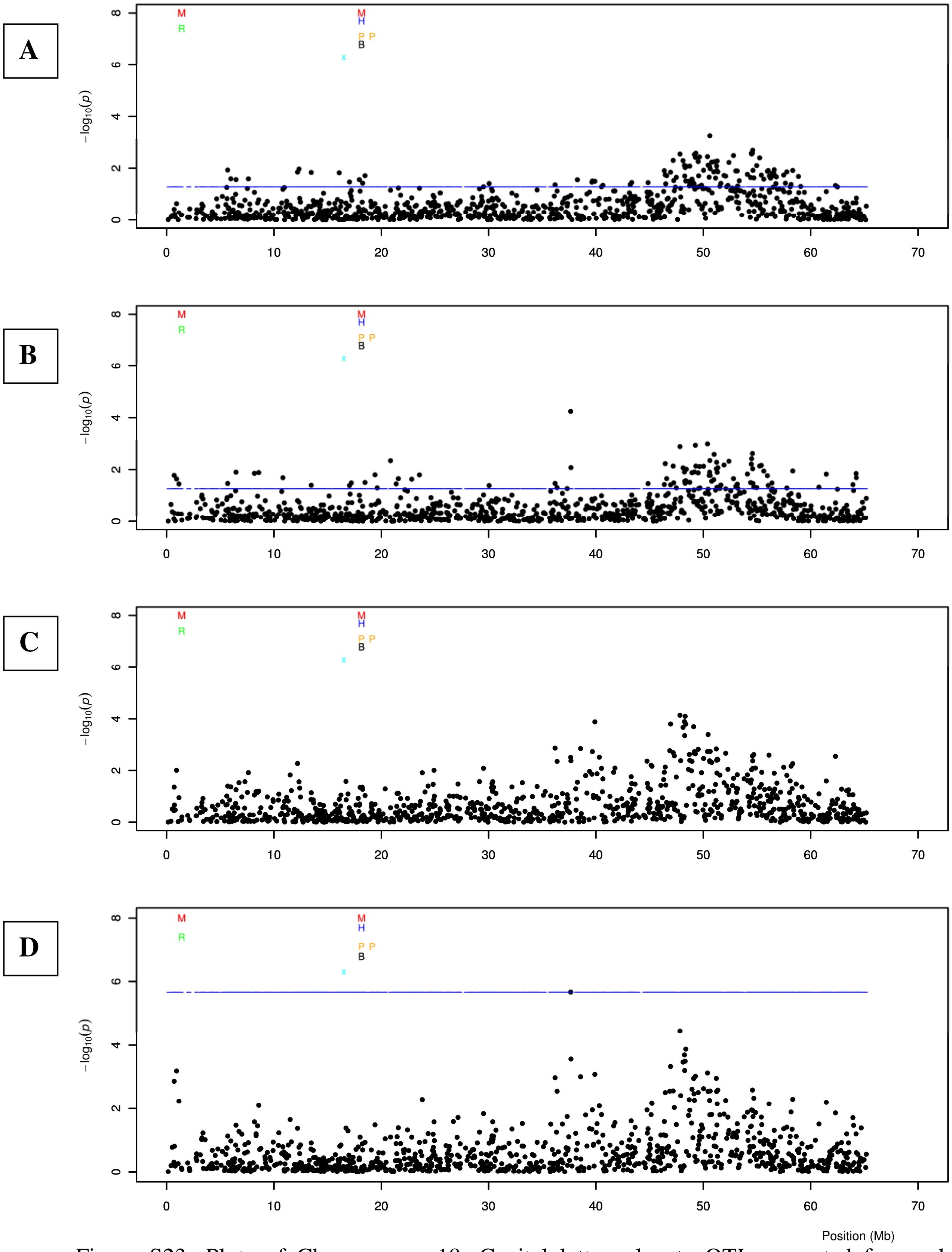

Figure S23: Plots of Chromosome 19; Capital letters denote QTLs reported from whole genome association studies (WGA) [52], summarized as QTL trait ontology classes: B.. meat traits, E... exterior traits, H.. health traits, M.. milk traits, P.. production traits, R.. reproduction traits; o annotates a top 5% iHS^{Voight} test statistic as reported in by [18] in windows of 500 kb in Brown Swiss, x in any of the other breeds investigated; Plot A: iHS^{Voight} test statistics, blue line: threshold identifying the top 5%; B: iHS test statistics, blue line: threshold identifying the top 5%; C: combined iHS^{Voight} and WGA results with model MIXstrat, D: combined iHS and WGA result with model MIXstrat; blue line is a at 10% false discovery rate threshold

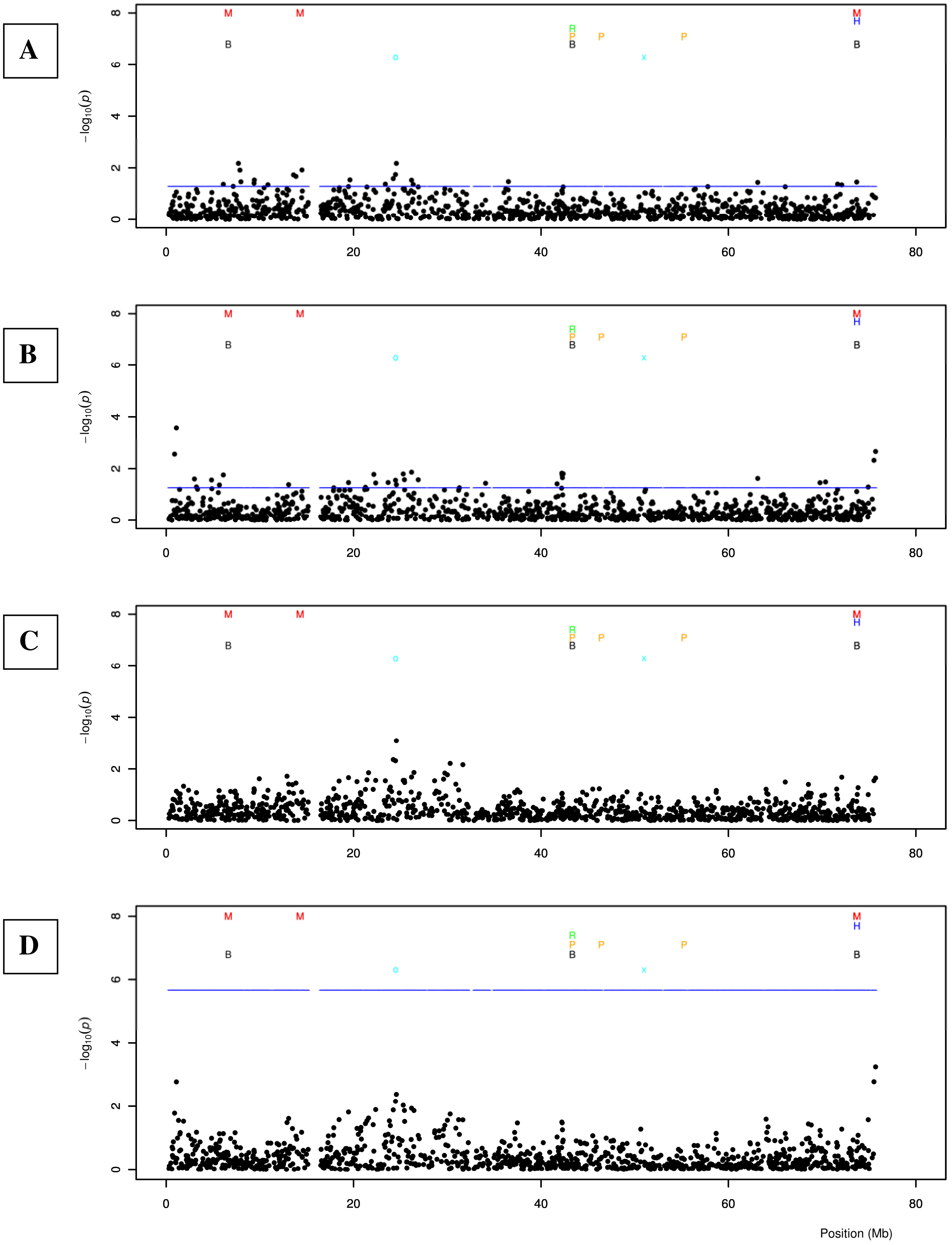

Figure S24: Plots of Chromosome 20; Capital letters denote QTLs reported from whole genome association studies (WGA) [52], summarized as QTL trait ontology classes: B.. meat traits, E... exterior traits, H.. health traits, M.. milk traits, P.. production traits, R.. reproduction traits; o annotates a top 5% iHS^{Voight} test statistic as reported in by [18] in windows of 500 kb in Brown Swiss, x in any of the other breeds investigated; Plot A: iHS^{Voight} test statistics, blue line: threshold identifying the top 5%; B: iHS test statistics, blue line: threshold identifying the top 5%; C: combined iHS^{Voight} and WGA results with model MIXstrat, D: combined iHS and WGA result with model MIXstrat; blue line is a at 10% false discovery rate threshold

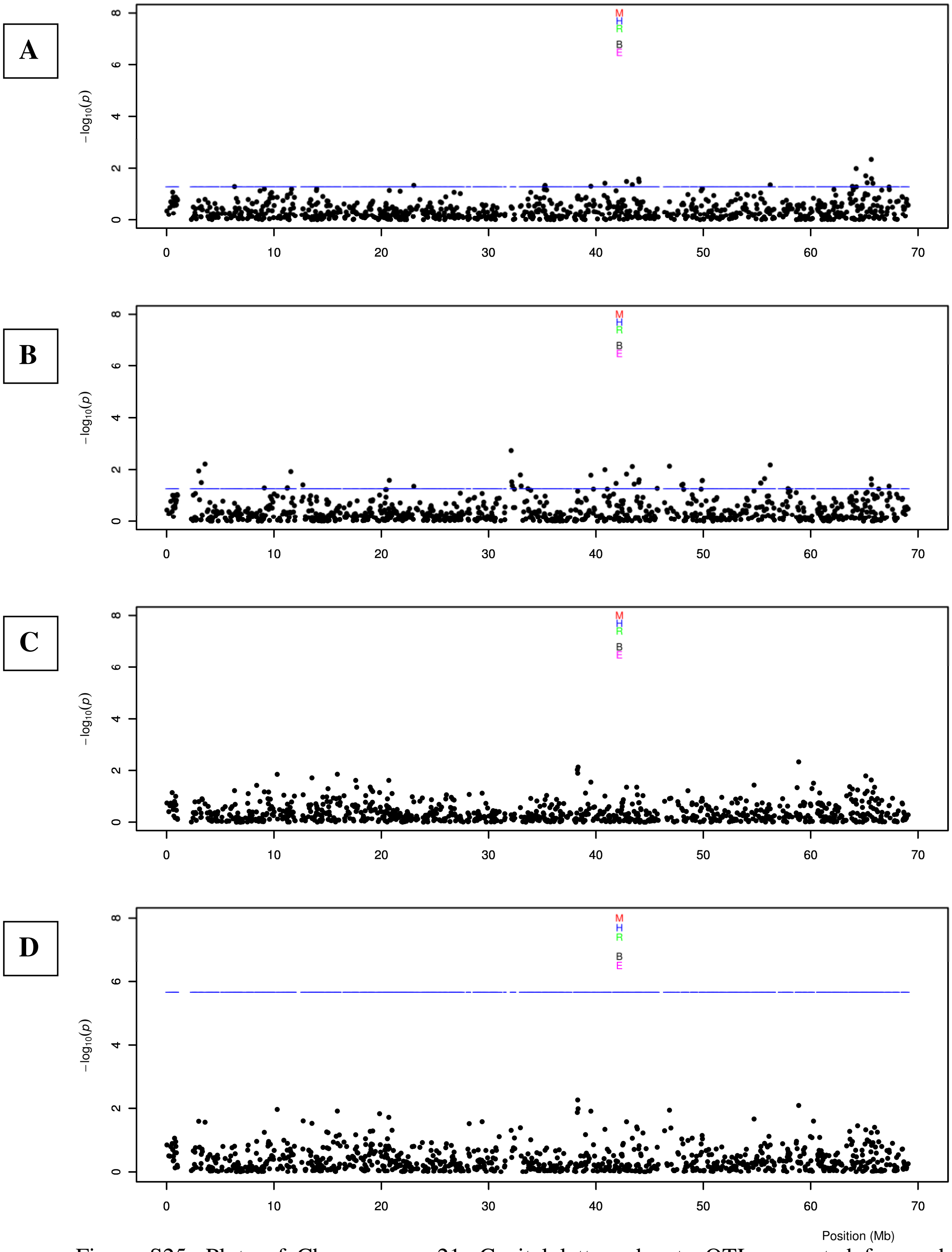

Figure S25: Plots of Chromosome 21; Capital letters denote QTLs reported from whole genome association studies (WGA) [52], summarized as QTL trait ontology classes: B.. meat traits, E... exterior traits, H.. health traits, M.. milk traits, P.. production traits, R.. reproduction traits; o annotates a top 5% iHS^{Voight} test statistic as reported in by [18] in windows of 500 kb in Brown Swiss, x in any of the other breeds investigated; Plot A: iHS^{Voight} test statistics, blue line: threshold identifying the top 5%; B: iHS test statistics, blue line: threshold identifying the top 5%; C: combined iHS^{Voight} and WGA results with model MIXstrat, D: combined iHS and WGA result with model MIXstrat; blue line is a at 10% false discovery rate threshold

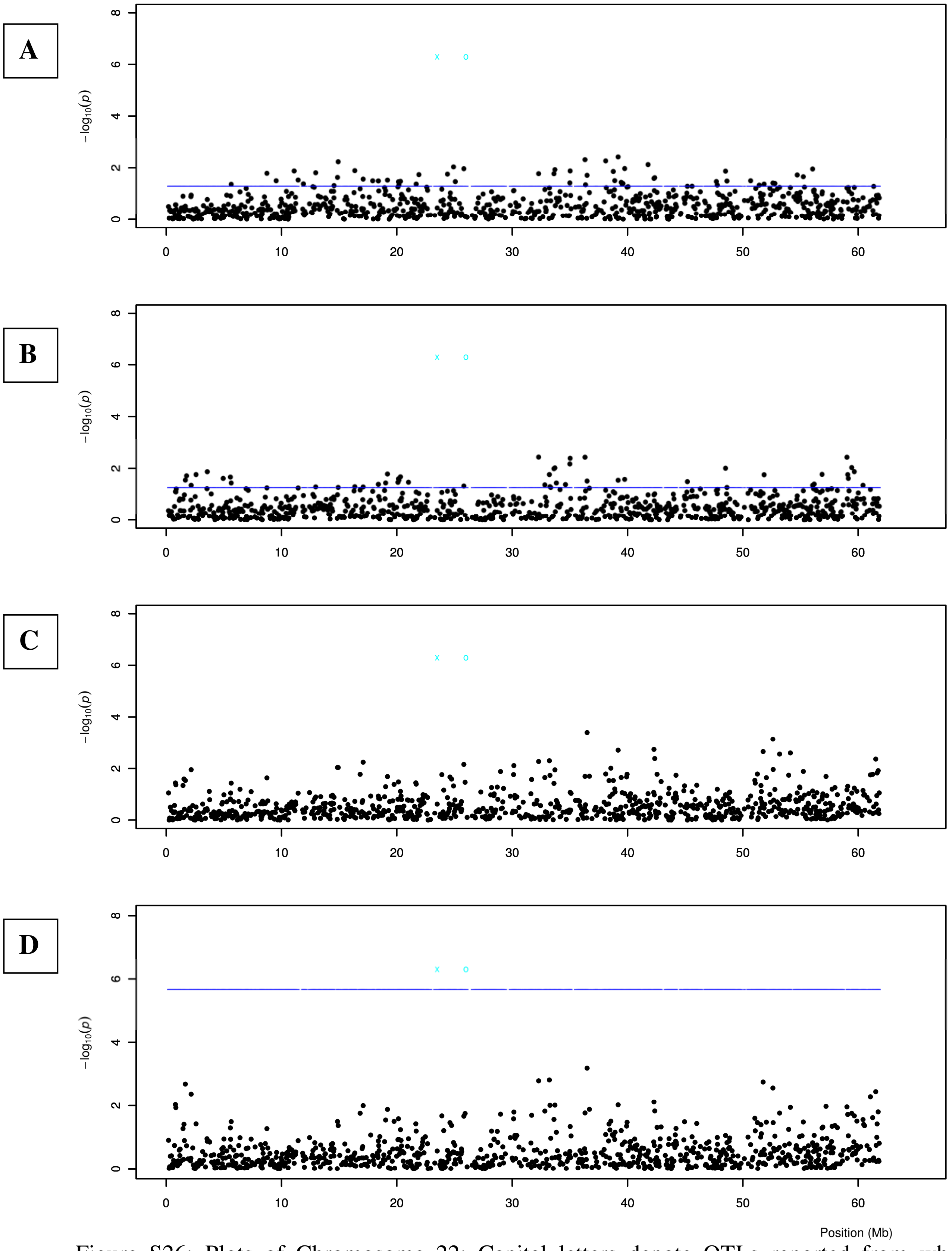

Figure S26: Plots of Chromosome 22; Capital letters denote QTLs reported from whole genome association studies (WGA) [52], summarized as QTL trait ontology classes: B.. meat traits, E... exterior traits, H.. health traits, M.. milk traits, P.. production traits, R.. reproduction traits; o annotates a top 5% iHS^{Voight} test statistic as reported in by [18] in windows of 500 kb in Brown Swiss, x in any of the other breeds investigated; Plot A: iHS^{Voight} test statistics, blue line: threshold identifying the top 5%; B: iHS test statistics, blue line: threshold identifying the top 5%; C: combined iHS^{Voight} and WGA results with model MIXstrat, D: combined iHS and WGA result with model MIXstrat; blue line is a at 10% false discovery rate threshold

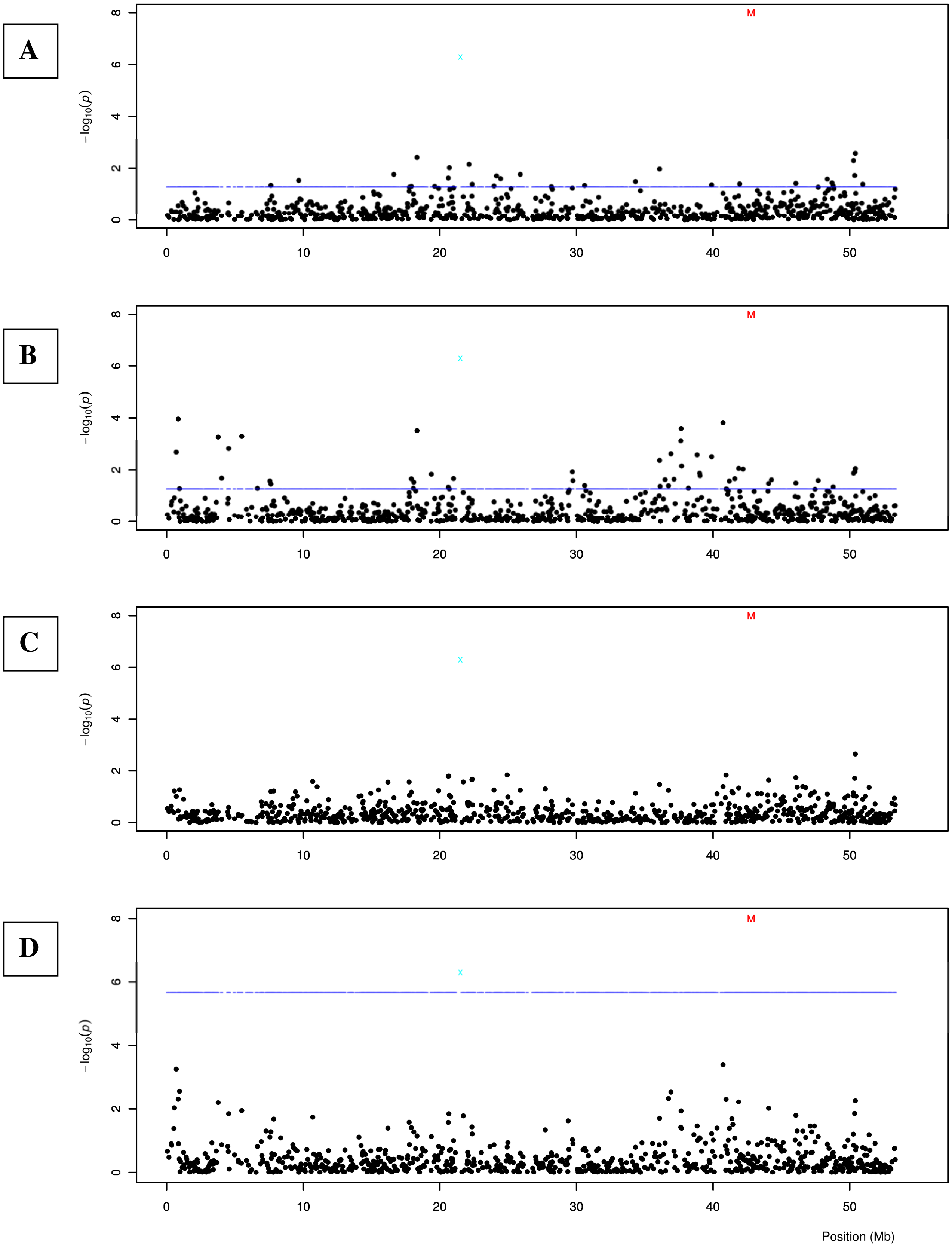

Figure S27: Plots of Chromosome 23; Capital letters denote QTLs reported from whole genome association studies (WGA) [52], summarized as QTL trait ontology classes: B.. meat traits, E... exterior traits, H.. health traits, M.. milk traits, P.. production traits, R.. reproduction traits; o annotates a top 5% iHS^{Voight} test statistic as reported in by [18] in windows of 500 kb in Brown Swiss, x in any of the other breeds investigated; Plot A: iHS^{Voight} test statistics, blue line: threshold identifying the top 5%; B: iHS test statistics, blue line: threshold identifying the top 5%; C: combined iHS^{Voight} and WGA results with model MIXstrat, D: combined iHS and WGA result with model MIXstrat; blue line is a at 10% false discovery rate threshold

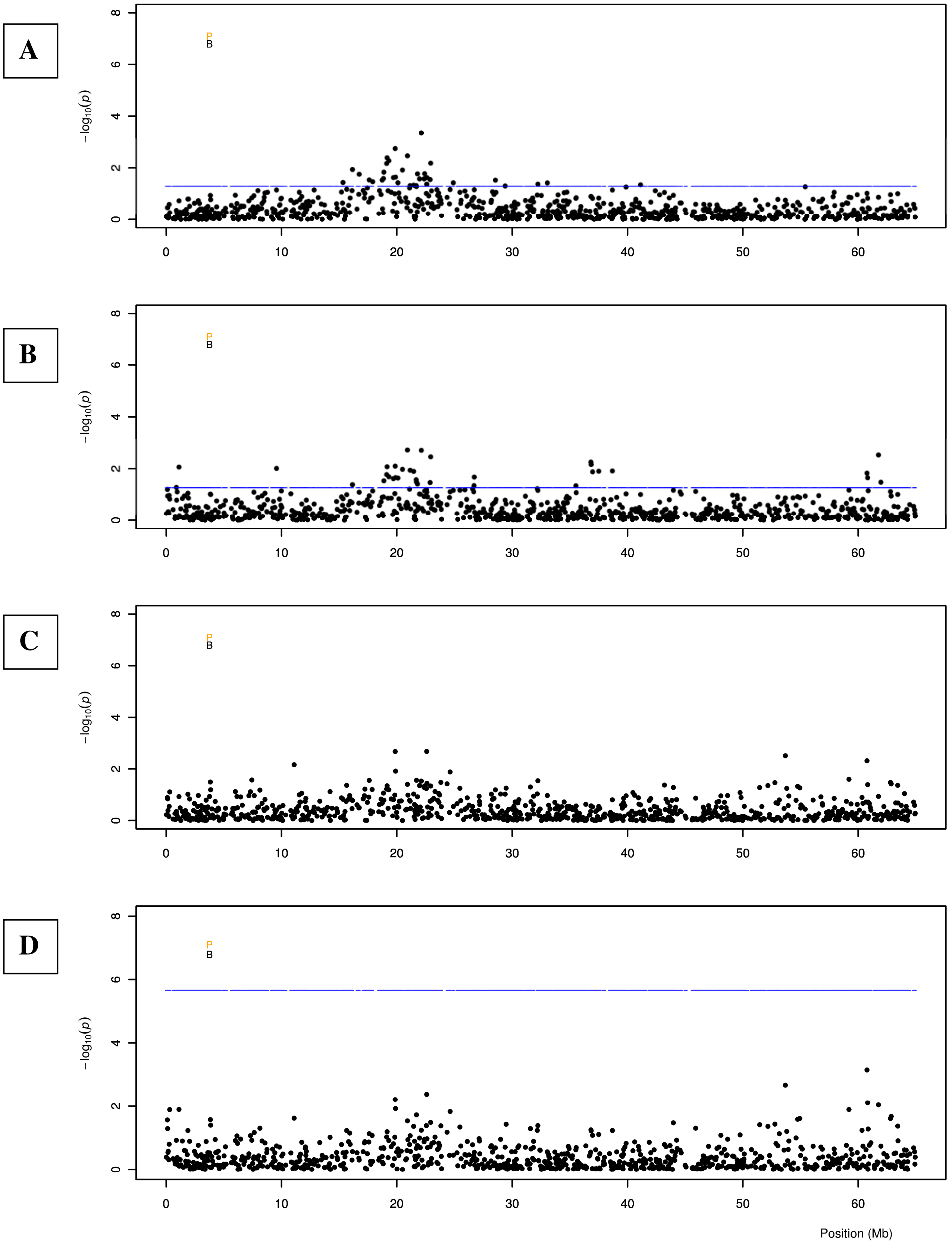

Figure S28: Plots of Chromosome 24, Capital letters denote QTLs reported from whole genome association studies (WGA) [52], summarized as QTL trait ontology classes: B.. meat traits, E... exterior traits, H.. health traits, M.. milk traits, P.. production traits, R.. reproduction traits; o annotates a top 5% iHS^{Voight} test statistic as reported in by [18] in windows of 500 kb in Brown Swiss, x in any of the other breeds investigated; Plot A: iHS^{Voight} test statistics, blue line: threshold identifying the top 5%; B: iHS test statistics, blue line: threshold identifying the top 5%; C: combined iHS^{Voight} and WGA results with model MIXstrat, D: combined iHS and WGA result with model MIXstrat; blue line is a at 10% false discovery rate threshold

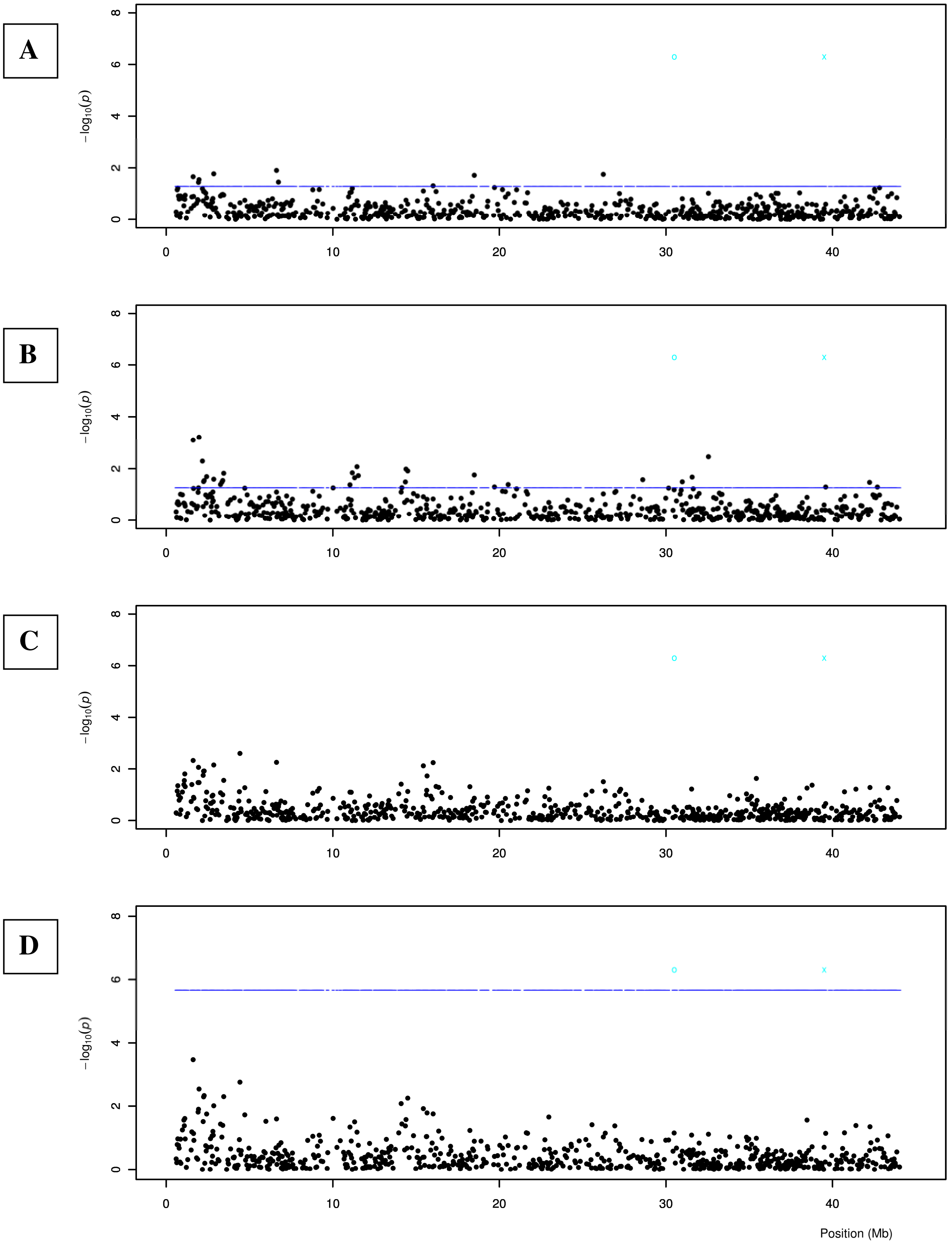

Figure S29: Plots of Chromosome 25; Capital letters denote QTLs reported from whole genome association studies (WGA) [52], summarized as QTL trait ontology classes: B.. meat traits, E... exterior traits, H.. health traits, M.. milk traits, P.. production traits, R.. reproduction traits; o annotates a top 5% $\text{iHS}^{\text{Voight}}$ test statistic as reported in by [18] in windows of 500 kb in Brown Swiss, x in any of the other breeds investigated; Plot A: $\text{iHS}^{\text{Voight}}$ test statistics, blue line: threshold identifying the top 5%; B: iHS test statistics, blue line: threshold identifying the top 5%; C: combined $\text{iHS}^{\text{Voight}}$ and WGA results with model MIXstrat, D: combined iHS and WGA result with model MIXstrat; blue line is a at 10% false discovery rate threshold

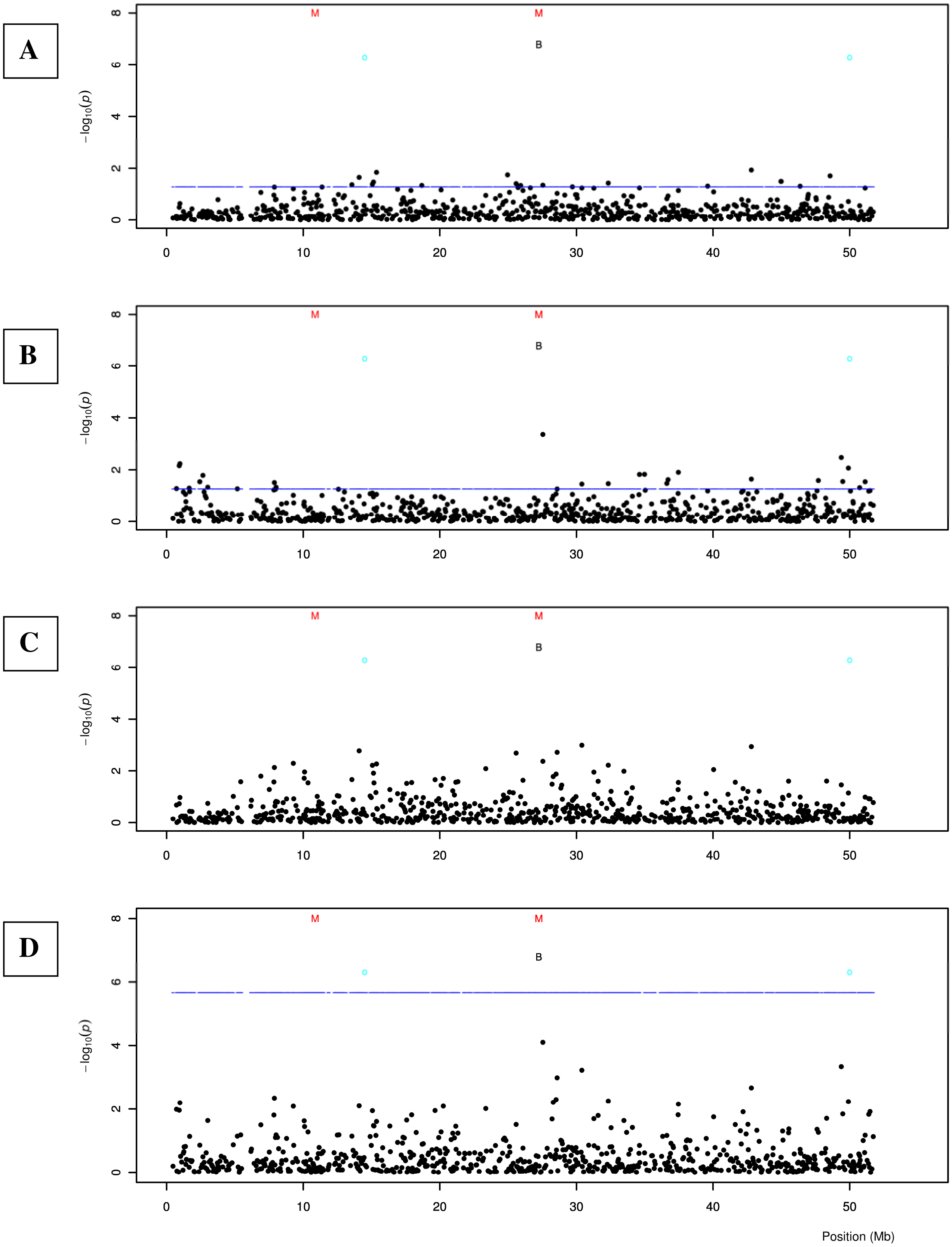

Figure S30: Plots of Chromosome 26; Capital letters denote QTLs reported from whole genome association studies (WGA) [52], summarized as QTL trait ontology classes: B.. meat traits, E... exterior traits, H.. health traits, M.. milk traits, P.. production traits, R.. reproduction traits; o annotates a top 5% iHS^{Voight} test statistic as reported in by [18] in windows of 500 kb in Brown Swiss, x in any of the other breeds investigated; Plot A: iHS^{Voight} test statistics, blue line: threshold identifying the top 5%; B: iHS test statistics, blue line: threshold identifying the top 5%; C: combined iHS^{Voight} and WGA results with model MIXstrat, D: combined iHS and WGA result with model MIXstrat; blue line is a at 10% false discovery rate threshold

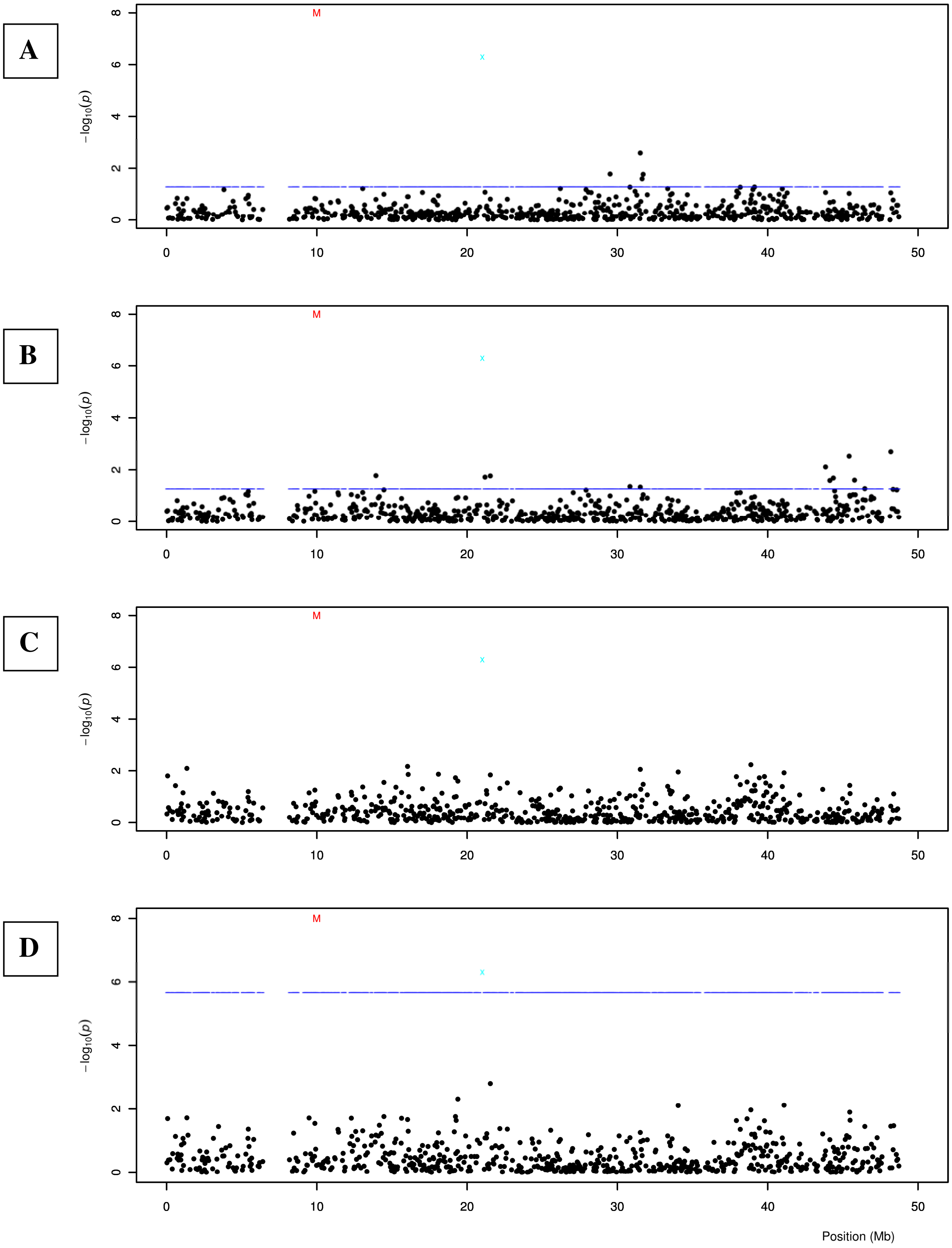

Figure S31: Plots of Chromosome 27; Capital letters denote QTLs reported from whole genome association studies (WGA) [52], summarized as QTL trait ontology classes: B.. meat traits, E... exterior traits, H.. health traits, M.. milk traits, P.. production traits, R.. reproduction traits; o annotates a top 5% iHS^{Voight} test statistic as reported in by [18] in windows of 500 kb in Brown Swiss, x in any of the other breeds investigated; Plot A: iHS^{Voight} test statistics, blue line: threshold identifying the top 5%; B: iHS test statistics, blue line: threshold identifying the top 5%; C: combined iHS^{Voight} and WGA results with model MIXstrat, D: combined iHS and WGA result with model MIXstrat; blue line is a at 10% false discovery rate threshold

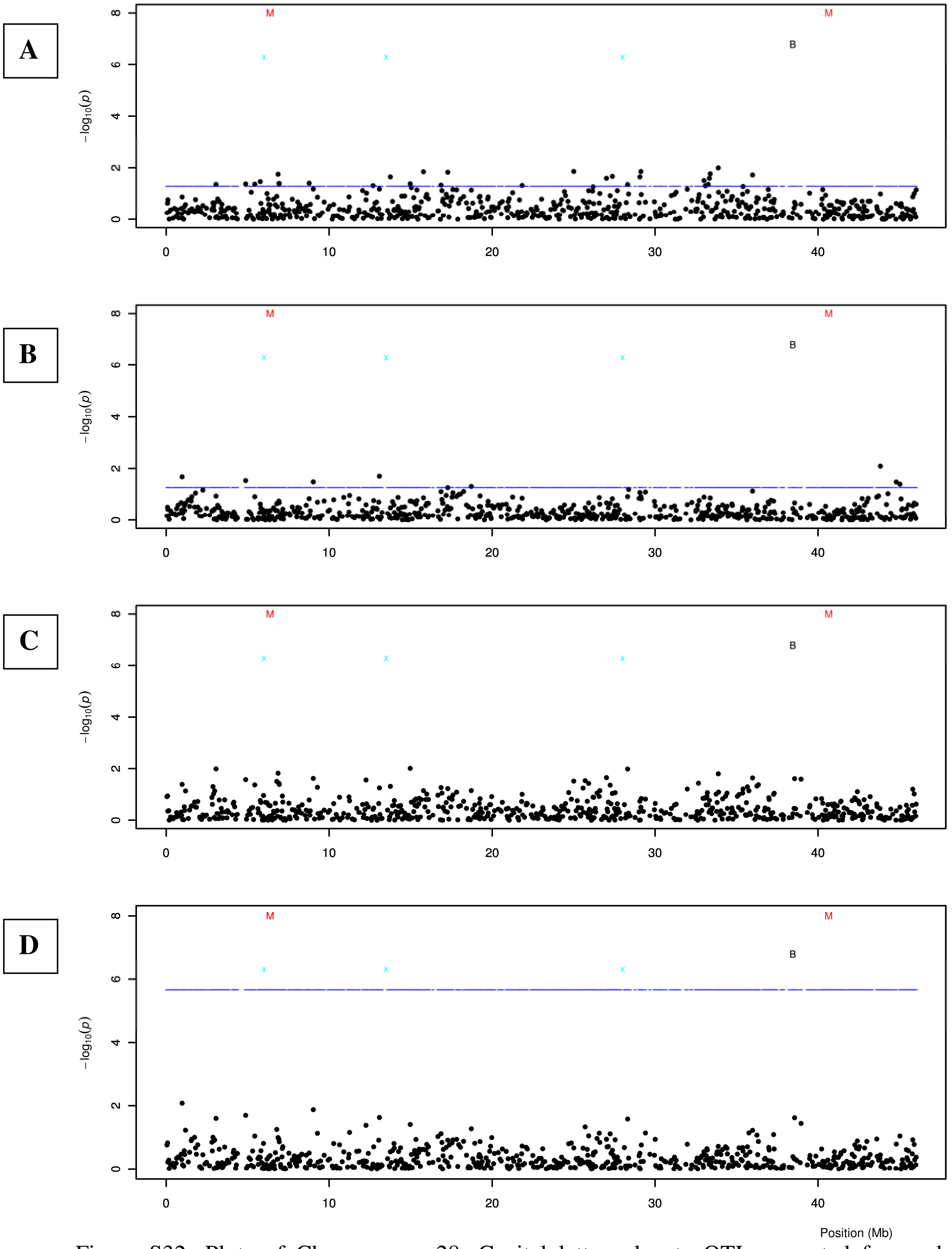

Figure S32: Plots of Chromosome 28; Capital letters denote QTLs reported from whole genome association studies (WGA) [52], summarized as QTL trait ontology classes: B.. meat traits, E... exterior traits, H.. health traits, M.. milk traits, P.. production traits, R.. reproduction traits; o annotates a top 5% iHS^{Voight} test statistic as reported in by [18] in windows of 500 kb in Brown Swiss, x in any of the other breeds investigated; Plot A: iHS^{Voight} test statistics, blue line: threshold identifying the top 5%; B: iHS test statistics, blue line: threshold identifying the top 5%; C: combined iHS^{Voight} and WGA results with model MIXstrat, D: combined iHS and WGA result with model MIXstrat; blue line is a at 10% false discovery rate threshold

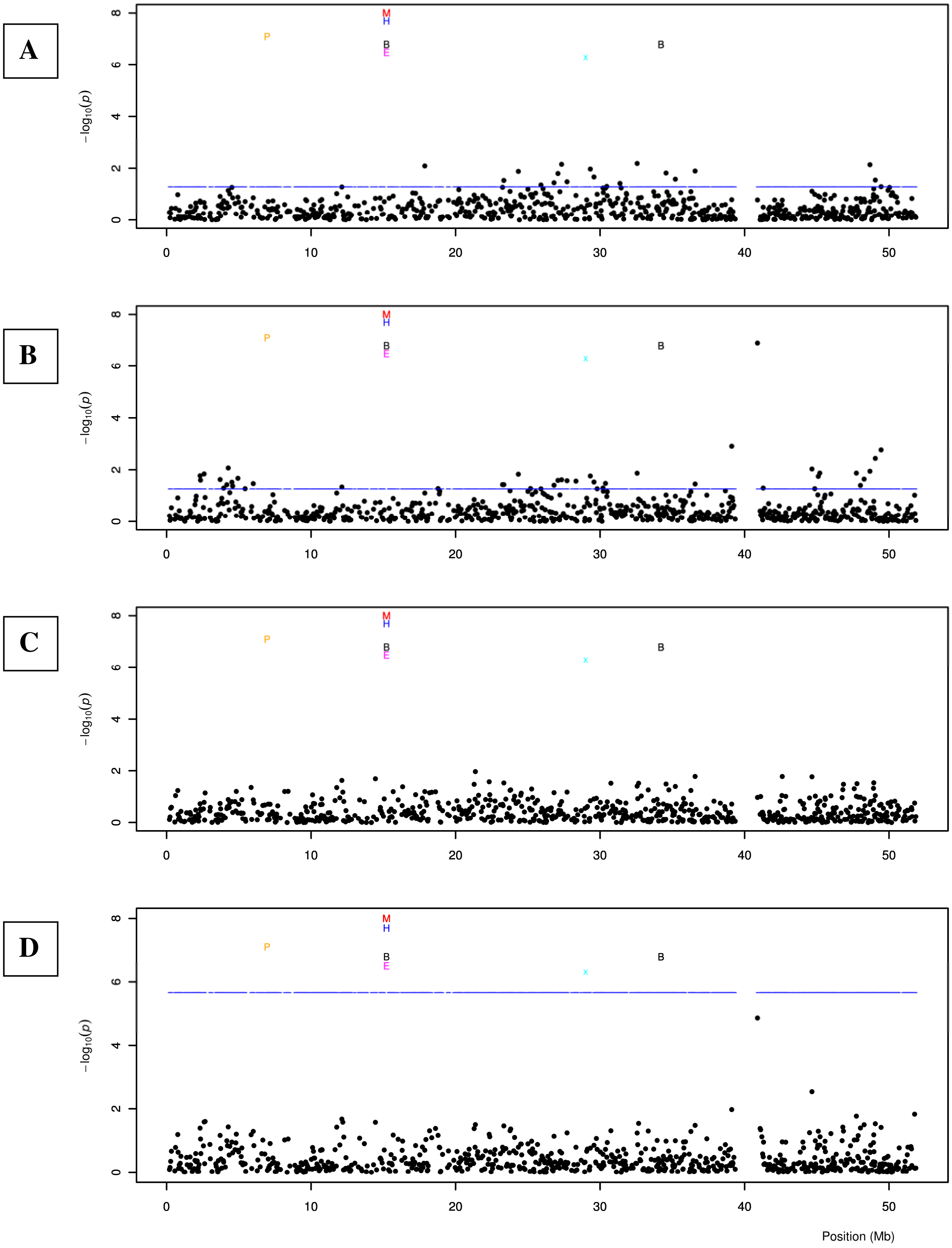

Figure S33: Plots of Chromosome 29; Capital letters denote QTLs reported from whole genome association studies (WGA) [52], summarized as QTL trait ontology classes: B.. meat traits, E... exterior traits, H.. health traits, M.. milk traits, P.. production traits, R.. reproduction traits; o annotates a top 5% iHS^{Voight} test statistic as reported in by [18] in windows of 500 kb in Brown Swiss, x in any of the other breeds investigated; Plot A: iHS^{Voight} test statistics, blue line: threshold identifying the top 5%; B: iHS test statistics, blue line: threshold identifying the top 5%; C: combined iHS^{Voight} and WGA results with model MIXstrat, D: combined iHS and WGA result with model MIXstrat; blue line is a at 10% false discovery rate threshold
